# Single-stranded binding proteins and helicase enhance the activity of prokaryotic argonautes *in vitro*

**DOI:** 10.1371/journal.pone.0203073

**Published:** 2018-08-29

**Authors:** Eric A. Hunt, Thomas C. Evans, Nathan A. Tanner

**Affiliations:** New England Biolabs, Inc., Ipswich, Massachusetts, United States of America; Saint Louis University, UNITED STATES

## Abstract

Prokaryotic argonautes are a unique class of nucleic acid-guided endonucleases putatively involved in cellular defense against foreign genetic elements. While their eukaryotic homologs and Cas protein counterparts require single-stranded RNAs as guides, some prokaryotic argonautes are able to utilize short single-stranded DNAs as guides for sequence-specific endonuclease activity. Many complications currently prevent the use of prokaryotic argonautes for *in vivo* gene-editing applications; however, they do exhibit potential as a new class of *in vitro* molecular tools if certain challenges can be overcome, specifically the limitations on substrate accessibility which leads to unequal levels of activity across a broad palate of substrates and the inability to act on double-stranded DNA substrates. Here we demonstrate the use of accessory factors, including thermostable single-stranded DNA binding proteins and UvrD-like helicase, in conjunction with prokaryotic argonautes to significantly improve enzymatic activity and enable functionality with a broader range of substrates, including linear double-stranded DNA substrates. We also demonstrate the use of *Thermus thermophilus* argonaute with accessory factors as a programmable restriction enzyme to generate long, unique single-stranded overhangs from linear double-stranded substrates compatible with downstream ligation.

## Introduction

Argonautes are nucleic acid-guided endonucleases generally divided into two main groups: eukaryotic argonautes (eAgos) and prokaryotic argonautes (pAgos). The eAgos are well known for their role in RNA interference (RNAi) pathways where they are a principle component of the RNA-induced silencing complex (RISC). Within the RISC, eAgos are involved in binding small RNA guides which are used as a directive template for various mechanisms of downstream post-transcriptional regulation and translational disruption of mRNA, depending on the level of sequence complementarity [1–3]. While the scope of eAgo activity with relation to RNAi comprises a broad, well-established field of study, pAgos, which are putatively involved in cellular defense against foreign and mobile genetic elements [3–6], exhibit a much broader range of enzymatic activity in relation to substrate and guide preferences.

Prokaryotic argonautes are very similar in structure to eAgos and are comprised of the same four domains (in order from N- to C-terminus): (1) the N domain, thought to play a key role in dissociation of cleaved substrate [3, 7, 8]; (2) the PAZ domain, responsible for binding the 3′ end of the guide nucleic acid [3, 9, 10]; (3) the MID domain, responsible for binding the 5′ end of the guide nucleic acid [3, 11, 12]; and (4) the PIWI domain, containing an RNase H-like active site [3, 13–15]. Considerable work has been done to elucidate the mechanisms by which argonautes—both eAgos and pAgos—target, bind, and interact with their substrates [14, 16–21]. Beyond the structural similarities, pAgos exhibit many marked differences from eAgos. Principally, pAgos are presumed to function in a more independent fashion. Capable of generating their own nucleic acid guides [22–27], pAgos may also act on substrate nucleic acids in the absence of multi-protein interactions observed with eAgos in the RISC. [3, 28, 29]. Additionally, some pAgos show strong preference to DNA guides rather than RNA guides, and act on DNA substrates rather than the mRNA substrates targeted by eAgos [3, 23, 25, 30].

This novel activity has led to many interesting and speculative applications for pAgos, e.g. programmable restriction enzymes [31] and tools for genome editing [6, 32]. The latter has been fraught with many critical issues regarding reproducibility and consensus on the nature of proposed enzymatic function [33–35]; however, pAgos have gained considerable attention and shown potential in becoming a new class of molecular tools, a trend paralleled by growing applications of Cas9 and other CRISPR-associated guided-nucleases beyond genome editing [36–40]. A potential boon to such applications, pAgos could be similarly applied, do not require a protospacer adjacent motif (PAM) to be included in the targeted sequence, and can utilize short, synthetic DNA guides in place of more expensive and harder-to-produce guide RNAs for CRISPR-Cas systems.

Argonautes have the ability to search for and act on their guide-defined substrate at a rate near the limit of diffusion [41], which is achieved in part through helical ordering of the seed region (nucleotides 2–8) of the short guide (typically <30 nt) thereby reducing the entropic barrier to duplex formation [41, 42]. While this interesting and powerful structural feature of argonautes allows for more rapid seeking of substrate nucleic acids, it requires the substrate to be exposed in a single-stranded form for binding and subsequent cleavage. These structural realizations suggest that potential improvements to binding and subsequent endonuclease activity could be made through the addition of accessory factors which ameliorate some of the initial steps that hinder argonaute activity.

Among other well studied pAgos, *Thermus thermophilus* argonaute (*Tt*Ago) is a DNA guided DNA endonuclease with a preference for short (16–22 nt) 5′-phosphorylated DNA guides [23]. The crystal structure for *Tt*Ago bound to a 21-mer DNA guide and 19-mer DNA substrate has been solved [20], and the initial enzymatic properties of *Tt*Ago have been well characterized [23, 26]. It has been reported that *Tt*Ago is able to cleave negatively supercoiled plasmid DNA, but it cannot act on linear double-stranded DNA (dsDNA) substrates [23]. This requirement for supercoiled structure is likely due to the resulting torsional strain which causes local clusters of duplex instability that are more accessible to argonaute [43, 44]. In eukaryotes, argonautes do not act alone, and require a host of other enzymatic partners to generate and load guides as well as carry out their diverse set of functions as a component of the RISC [3, 28, 29]. Cursory exploration of the open reading frames surrounding pAgos reveals that accessory factors, such as helicases or other nucleases, are frequently found within the same operon [3, 6, 30]. These and other accessory factors could aid pAgos in their native *in vivo* function.

Helicases are proteins which actively couple ATP hydrolysis to unwinding of and translocation on nucleic acid substrates [45]. UvrD helicases are a well studied group of superfamily 1 helicases found in almost all bacteria and are typically involved in repair pathways, such as double-strand break repair where they interact with other proteins such as nucleases in this crucial process [46]. Like helicases, single-stranded DNA binding proteins (SSBs) are also involved in (among many cellular activities) replication, recombination, repair, and transcription of nucleic acids [47, 48]. Many are dynamic in binding allowing other proteins to interact with the bound ssDNA [49] and some have been shown to have direct interactions via an acidic C-terminal region with various other protein components to carry out these processes [50–52]. While there is wide sequence variability among this type of protein, the oligonucleotide-/oligosaccharide-binding (OB) fold is a fairly common, well-studied component among SSBs, and is capable of binding ssDNA and ssRNA, in addition to a wide variety of other nucleic acid moieties, in a non-sequence-dependent manner [53]. Through this binding, some SSBs are able to melt low-stability or AT-rich regions of dsDNA and can propagate duplex unwinding by destabilizing the helix-structure of dsDNA in an ATP-independent fashion [49, 50, 52].

As SSBs and helicases have been shown to interact with eAgos [28], we were curious to explore potential relationships between these accessory factors and pAgos, and we hypothesized that highly thermostable variants could potentially improve *in vitro* functionality of pAgos. Herein we present the use of thermostable SSBs and *Thermus thermophilus* helicase (*Tth*UvrD) [54] and their effects on the activity of pAgos from *Thermus thermophilus* (*Tt*Ago), *Pyrococcus furiosus* (*Pf* Ago), and *Natronobacterium gregoryi* (*Ng*Ago). We have found that the addition of accessory factors significantly enhances the activity of *Tt*Ago *in vitro*, enabling catalytic activity on substrates, specifically dsDNA and high GC content single-stranded DNA (ssDNA), which otherwise support low or no cleavage by *Tt*Ago. Furthermore these accessory factors enhance the specificity of guide-substrate binding, enabling stringent single-nucleotide discrimination within the supplementary region (nucleotides 12–15) of the guide, and expand the range of DNA guide lengths compatible with efficient substrate cleavage. Improved reliability and expanded substrate accessibility represent steps toward realizing pAgos as molecular tools.

## Results and discussion

### Thermostable helicase *Tth*UvrD improves *Tt*Ago activity on plasmid substrates

**Fig 1:**
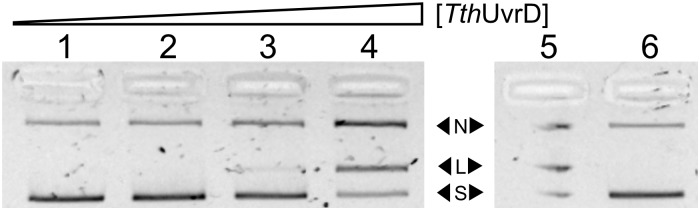
Effect of *Tth*UvrD on *Tt*Ago activity *in vitro*. Addition of *Tth*UvrD improves *Tt*Ago activity on dsDNA pUC19 plasmid substrate cut with guides pUC19-1 and pUC19-2 (Supporting information S1 Table). Reactions were supplemented with 1 mM ATP and carried out at 73 °C for 4 hr. Lanes 1–4) 10, 25, 50, and 100 ng of helicase, respectively; 5) pUC19 controls: nicked (N) with Nt.BspQI, linearized (L) with SspI, and supercoiled (S); 6) no helicase control—some plasmid relaxation occurs during prolonged incubation at high temperatures.

As many UvrDs are well characterized and are functional on a variety of different substrates, including blunt dsDNA substrates and nicked/relaxed plasmid DNA substrates [55], we investigated the effects of UvrD-like helicases on *Tt*Ago activity. We observed that the addition of thermostable *Tth*UvrD to the reaction enhanced the ability of *Tt*Ago to cut a plasmid substrate in 4 hr using two guides targeting complementary strands to generate a double-strand break (Fig 1). *Tth*UvrD is similar to *E. coli* helicase II UvrD [56] and is able to unwind a broad range of substrates, including blunt-end duplex DNA [55].

*Tt*Ago has been reported to generate its own guides from dsDNA; however, it is unable to act on double-stranded regions of elevated GC content or stability, and instead is dependent upon inherently unstable regions resulting from, for example, low GC content, active transcription, or torsional strain on the duplex from supercoiling [26, 43, 44]. From an evolutionary standpoint, it is reasonable to consider that in bacteria and archaea, as is observed in eukaryotes, iterative pathways have emerged in which accessory proteins interact with pAgos to enable or regulate their *in vivo* functionality. The exploration of open reading frames within the same operons containing pAgos reveals that they likely do not function alone [6, 23], though as previously described they do exhibit some autonomous ability beyond what is observed with eAgos [26, 27]. These potential functional partners have been identified as helicases, nucleic acid binding proteins, and other restriction endonuclease s. We reasoned that the inclusion of some of these functional partners or similarly functioning accessory proteins may improve pAgo activity on a broader set of substrates *in vitro*.

Interestingly, several different UvrD-like helicases from bacterial and archaeal thermophiles did not stimulate *Tt*Ago activity—only *Tth*UvrD affected argonaute activity. While this may be in part due to other helicases having different *in vitro* buffer preferences, it is also possible that some interaction may occur between the helicase and *Tt*Ago [28]. Controls containing *Tth*UvrD and ET SSB only with plasmid substrate were carried out at 74 °C for 8 hr to verify that activity observed was not due to contaminating endonuclease (Supporting information S5B–S5C Fig). Removal of ATP from the reaction buffer precluded this effect on *Tt*Ago activity (Supporting information S5D Fig). Additionally, the four catalytic residues (D478, E512, D546, and D660) involved in the RNase H-type active site of *Tt*Ago [20] were individually mutated to alanine. Mutation of any of these residues rendered *Tt*Ago inactive and no guided or unguided nuclease activity was observed when compared to the native sequence (Supporting information S8 Fig) providing further evidence that contaminant nucleases were not responsible for the guided nuclease activity observed for purified *Tt*Ago.

### ET SSB with *Tt*Ago reduces substrate GC content preference and enables activity on linear dsDNA substrate

**Fig 2:**
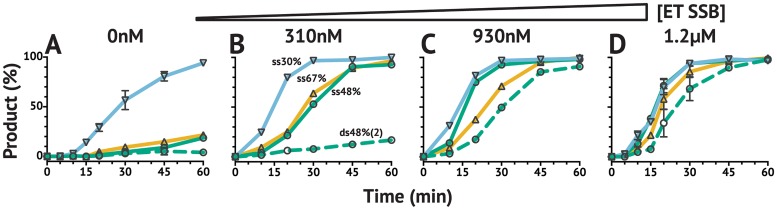
Effect of ET SSB on *Tt*Ago activity *in vitro* using 21 nt guides. *Tt*Ago exhibits strong preference for substrates with lower GC content. This has been reported for dsDNA plasmids [3, 26], but was also observed for ssDNA substrates. The addition of ET SSB allowed for equivalent *Tt*Ago activity on ssDNA and dsDNA substrates with GC contents ranging from 30–70%. (**A–D**) show *Tt*Ago activity at increasing concentrations of ET SSB. Single-stranded (ss) and double-stranded (ds; dashed line) substrates along with the corresponding GC contents (▽30%; ○48%; △67%) are labeled in (**B**). Percent product was determined as the ratio of product to substrate monitored by CE throughout the reaction.

**Fig 3:**
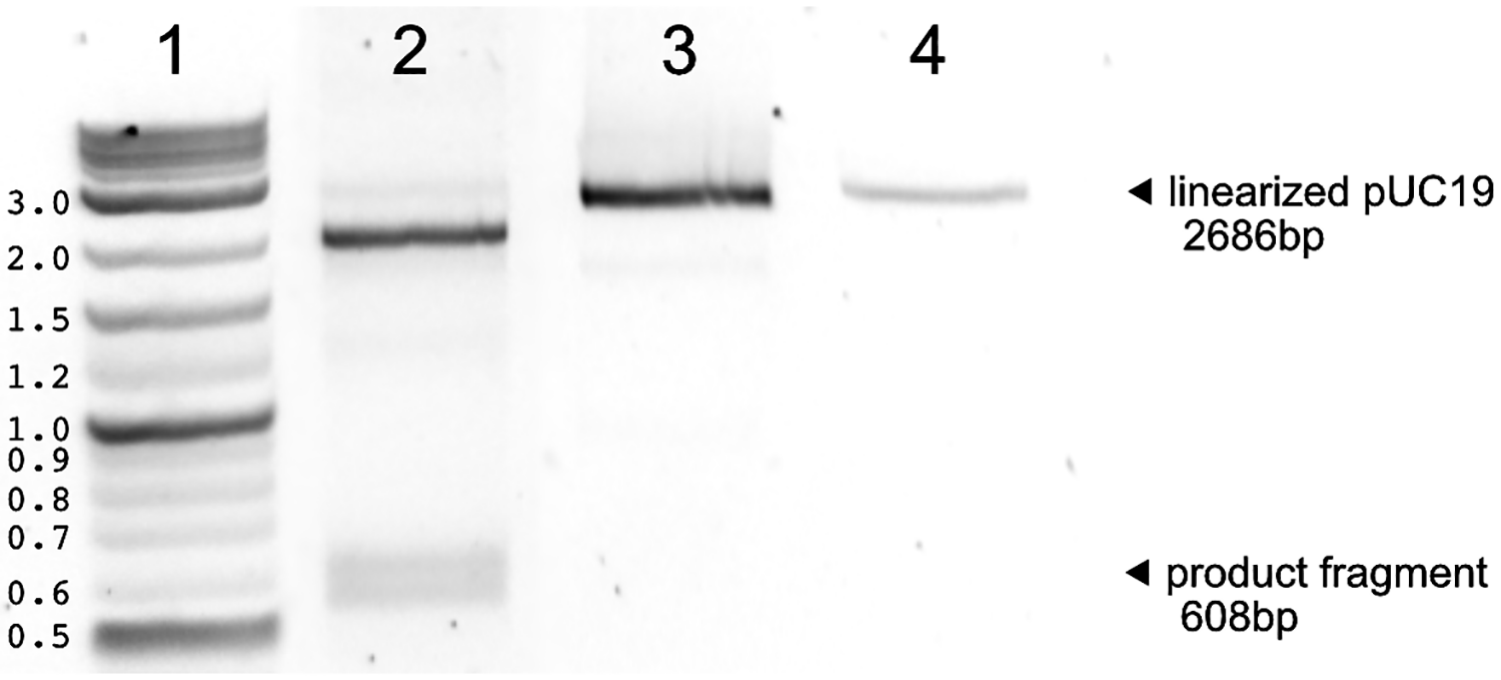
*Tt*Ago can cut linearized pUC19 in the presence of ET SSB. *Tt*Ago is able to cleave linear dsDNA in the presence of ET SSB. pUC19 (2686 bp) was linearized with SspI and used as a substrate for the reaction. Guides pUC19-1 and pUC19-2 (Supporting information S1 Table) were designed to cut near the BamHI restriction site on pUC19 to generate a 608 bp fragment. Lane 1) NEB Quick-Load Purple 2-Log DNA Ladder (0.1–10.0 kb); 2) *Tt*Ago + ET SSB; 3) *Tt*Ago only; 4) SspI linearized pUC19 control.

*Tt*Ago exhibits a strong preference for substrates with lower GC content (Fig 2A). This trend was observed even for ssDNA substrates—perhaps due to slower release of cleaved high GC content product from the guide, resulting in lower turnover and minimal product generation over a 2 hr incubation (data shown in Fig 2 is limited to 60 min). However, the addition of ET SSB dramatically reduced this substrate preference, and provided equivalent activity on substrates with GC content ranging from 30–70%GC (Fig 2B–2D). Additionally, ET SSB enabled the same level of activity on a 50%GC linear dsDNA substrate (Fig 2D). In reactions with *Tt*Ago alone, only a 30%GC substrate resulted in >50% cleavage in 2 hr, but with 1.2 *μ*M ET SSB (∼40 times excess of *Tt*Ago) all ssDNA substrates (30–70%GC) and the dsDNA substrate (50%GC) resulted in complete cleavage in 60 min or less. This activity was observed with fluorescent dye-labeled substrates analyzed by capillary electrophoresis (CE) (Fig 2) and with pUC19 plasmid linearized with SspI-HF (Fig 3). The addition of ET SSB assisted *Tt*Ago in generating a double-strand break into linearized pUC19 plasmid when provided with two guides targeting complementary strands. The pUC19 substrate had been linearized with SspI-HF prior to the reaction in order to generate a final product of known size (∼600 bp), which was then visualized by electrophoresis on a 1% agarose gel (Fig 3). This is a significant advancement as *Tt*Ago alone had not previously been demonstrated to be active on locally nicked/relaxed or linearized dsDNA substrates [23].

ET SSB is a single-stranded DNA binding protein with high thermostability, making it compatible with pAgos which function at elevated temperature, such as *Tt*Ago (∼75 °C) and *Pf* Ago (∼90 °C). ET SSB has been used to improve PCR and other amplification based methods, reducing non-specific amplification presumably by hampering secondary structure formation and promoting primer hybridization with only fully complementary sites [57–60]. Recently, SSBs were also demonstrated to facilitate primer invasion and suppress non-specific amplification in isothermal amplification methods [61]. Given these previous applications, SSBs could be beneficial when used with pAgos by eliminating secondary structure and potentially melting more stable duplex regions so that pAgo can search for, bind, and act more efficiently on a variety of substrates.

GC content variability between guides was achieved by varying the GC content in the seed and mid regions of the guide only—the supplementary and tail remained constant for all guides used in CE assays (see Supporting information S1 Table). As such, variability seen in activity on single-stranded substrates with different GC content is potentially due to seed binding or the release of the corresponding seed-mid region of the product rather than the corresponding supplementary/tail portion. While it is proposed that the N domain is involved with product and/or passenger strand release though a wedging process disrupting the hybridization [7, 8], ET SSB may be playing some role in this release process as well.

### ET SSB with *Pf* Ago increases reaction rates

**Fig 4:**
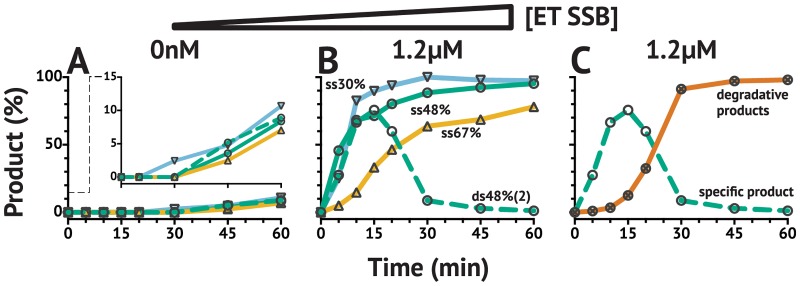
Effect of ET SSB on *Pf* Ago activity *in vitro* using 21 nt guides. The addition of ET SSB to *Pf* Ago increases its rate of activity, but does not affect GC content preference or ability to cleave dsDNA substrates. This is likely due to the reaction being performed at 90 °C where duplex and secondary structure are melted. (**A**) *Pf* Ago activity in the absence of ET SSB, and (**B**) *Pf* Ago activity in the presence of 1.2 *μ*M ET SSB. (**B–C**) The decrease in 50%GC dsDNA substrate after 15 min is due to degradation of the product. This only occurs with *Pf* Ago on dsDNA substrate, and is reminiscent of the apo-form degradation previously described for other pAgos [26, 27]. Single-stranded (ss) and double-stranded (ds; dashed line) substrates along with the corresponding GC contents (▽30%; ○48%; △67%) are labeled in (**B**). Degradative products are represented by the solid line with ⨂ markers in (**C**).

The addition of ET SSB significantly increased the reaction rate for *Pf* Ago, however there was no effect on GC content preference (Fig 4). Without ET SSB, only about 15% cleavage could be achieved with any substrate in 90 min, but addition of 1.2 *μ*M ET SSB (∼40 times excess of *Pf* Ago) enabled >80% cleavage, with the lower GC content substrates nearing completion in <30 min. Beyond this rate increase, there was no further beneficial effect observed for dsDNA substrates likely because reaction conditions—*Pf* Ago is active at ∼90 °C—do not allow for the substrate to remain in duplexed state. In previous publications describing the activity of *Pf* Ago, divalent manganese was used in the reaction buffer as opposed to divalent magnesium used here. When using manganese in the methods described herein, more aberrant activity and off-target cutting was observed for *Pf* Ago. For this reason, and to better compare the effects of ET SSB on *Pf* Ago to *Tt*Ago, reactions conditions were held constant between the two pAgos and divalent magnesium was used for both.

While addition of ET SSB to *Pf* Ago increased the rate of product formation, it also increased the amount of non-specific endonuclease activity observed, albeit only with dsDNA substrate. Given enough time, the substrate and expected specific product are completely degraded by *Pf* Ago into smaller fragments. This accounts for the decrease in product formation observed after 15 min (Fig 4B). CE results confirmed the generation of several smaller fragments coinciding with the disappearance of sequence-specific product (Fig 4C). The addition of higher stoichiometric equivalents of guide did not reduce the amount of non-specific activity observed (Supporting information S6 Fig), and the same non-specific activity was observed on both the 5′-FAM/3′-TAMRA and 5′-FAM/5′-HEX labeled substrates (Supporting information S6 Fig and S2 Table). Results with the latter substrate suggest the non-specific activity is not necessarily dependent upon an exposed 5′-phosphate, which would be blocked by the fluorescent dye-label. While it could be related to an apo-*Pf* Ago chopping activity as observed with other pAgos [26, 27], the inability of higher stoichiometric equivalents of guide to suppress off target cutting by apo-*Pf* Ago suggest it may be an inherent functionality not entirely dependent on the apo-form, i.e. the guide may easily dissociate from *Pf* Ago at high-temperatures. Recent computational work by Zhu et al. proposed an induced-fit model for guide loading in *Tt*Ago with a significant energy barrier to achieving a conformation amenable to guide loading [62]. If a similar model is true for other pAgos such as *Pf* Ago, the high operational temperature could cause *Pf* Ago to be in a more rapid flux between bound and unbound states due to the excess thermal energy present in the system, and could be another reason behind the high amount of degradation observed on dsDNA substrates.

### ET SSB enables *Tt*Ago to use a broader range of guide sizes

**Fig 5:**
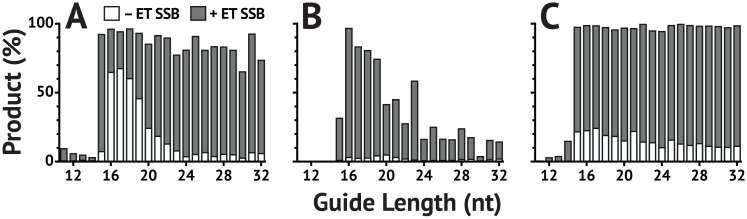
ET SSB enables the use of longer 5′-phosphorylated guides. *Tt*Ago can utilize 5′-phosphorylated guides of varying length, the most active falling in the range of 16–19 nt in the absence of ET SSB. (**A–C**) show percent product, determined by CE, after 1 hr at 73 °C either with 1.2 *μ*M ET SSB (dark grey) or without ET SSB (white). (**A**) *Tt*Ago with a single guide and ssDNA substrate; (**B**) *Tt*Ago with a single guide and dsDNA substrate; (**C**) *Pf* Ago with a single guide and ssDNA substrate.

**Fig 6:**
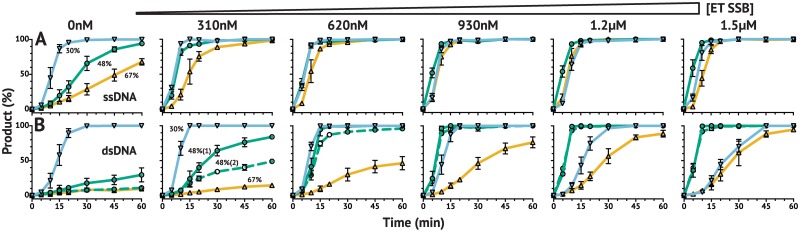
Effect of ET SSB on *Tt*Ago activity *in vitro* using 17 nt guides. Experiments shown in Fig 2 were repeated with more optimal 17 nt guides and expanded to include dsDNA substrates of 30%GC and 67%GC content, as well as a second dsDNA substrate of 48%GC content containing different fluorescent dye-labels (see S2 Table). The addition of ET SSB allowed for equivalent *Tt*Ago activity on ssDNA and dsDNA substrates with GC contents ranging from 30–70%. (**A**) shows *Tt*Ago activity on ssDNA substrates over increasing concentrations of ET SSB; corresponding GC contents (▽30%; ○48%; △67%) are labeled in the first panel from the left. (**B**) shows *Tt*Ago activity on dsDNA substrates over increasing concentrations of ET SSB; corresponding GC contents (▽30%; ○48%; △67%) are labeled in the second panel from the left, and the alternate 48%GC content substrate is represented by the solid green line to adhere to the labeling of Fig 2. Percent product was determined as the ratio of product to substrate monitored by CE throughout the reaction.

*Tt*Ago utilizes short 5′-phosphorylated ssDNA guides to target its substrate. These guides typically range from 16–22 nt in length, with the shorter guides (16–18 nt) being more effective than the longer guides (Fig 5A) in the absence of ET SSB. With the addition of ET SSB, however, a broader range of guide lengths—15 nt up to 32 nt (longer guides were not tested)—could be used to cut ssDNA and dsDNA substrates with *Tt*Ago (Fig 5A–5B). Native *Pf* Ago without ET SSB utilized 5′-phosphorylated ssDNA guides ranging from 15–32 nt, however, the addition of ET SSB increased activity for all guides in this 15–32 nt range (Fig 5C). The ability to use a broader range of guide lengths in the presence of ET SSB and still attain efficient activity from *Tt*Ago and *Pf* Ago on ssDNA and dsDNA targets simplifies the procedural complexity of using pAgos as molecular tools. To demonstrate this, we repeated the experiments shown in Fig 2, which used 21 nt guides, using more optimally sized 17 nt guides (Fig 6). Though the reaction was slightly faster using 17 nt guides, reactions went to completion with either length guide within 30 min in the presence of ET SSB.

### ET SSB limits *Tt*Ago sensitivity to mismatches within the supplementary region of the guide

**Fig 7:**
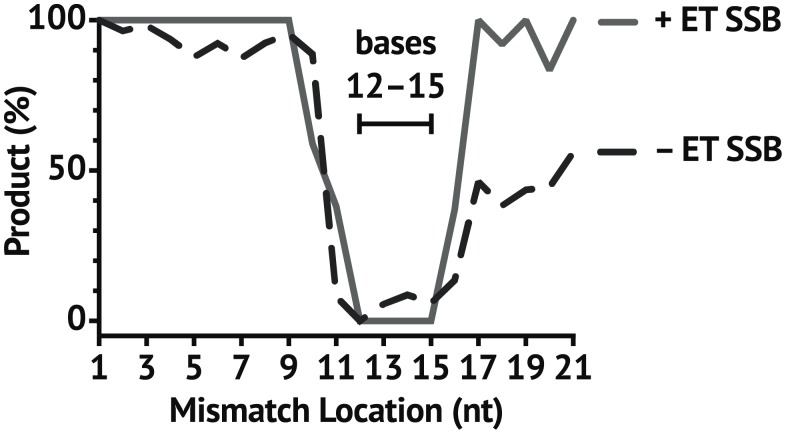
Effect of ET SSB on single-nucleotide mismatches within the guide. ET SSB increased *Tt*Ago sensitivity to mismatches in specific locations. ET SSB generally increased the trend of *Tt*Ago sensitivity to mismatches falling within the 12–15 region of the guide (supplementary region). Mismatches in the tail region had more of an inhibitory effect in the absence of ET SSB, and mismatches in the seed region had minimal effect on whether or not substrate was cleaved both with and without ET SSB. Percent product was determined as the ratio of product to substrate monitored by CE following incubation at 73 °C for 1 hr.

Argonautes segregate their short guides into four functional regions [42]. Single nucleotide mismatches in the seed region (nucleotides 2–8), while they could affect the rate of action on a substrate, did not affect the generation of final cleavage product (Fig 7). Mismatches occurring from nucleotides 12–15 have an especially significant impact on endonuclease activity with and without ET SSB present. However, the addition of ET SSB to *Tt*Ago reduced activity to zero for mismatches falling within this supplementary region of the guide as compared to a low level of cleavage activity observed without ET SSB. Additionally, mismatches falling within the tail region did not affect activity when ET SSB was present (Fig 7), which coincides with the previously mentioned ability to utilize longer guides (Fig 5).

**Fig 8:**
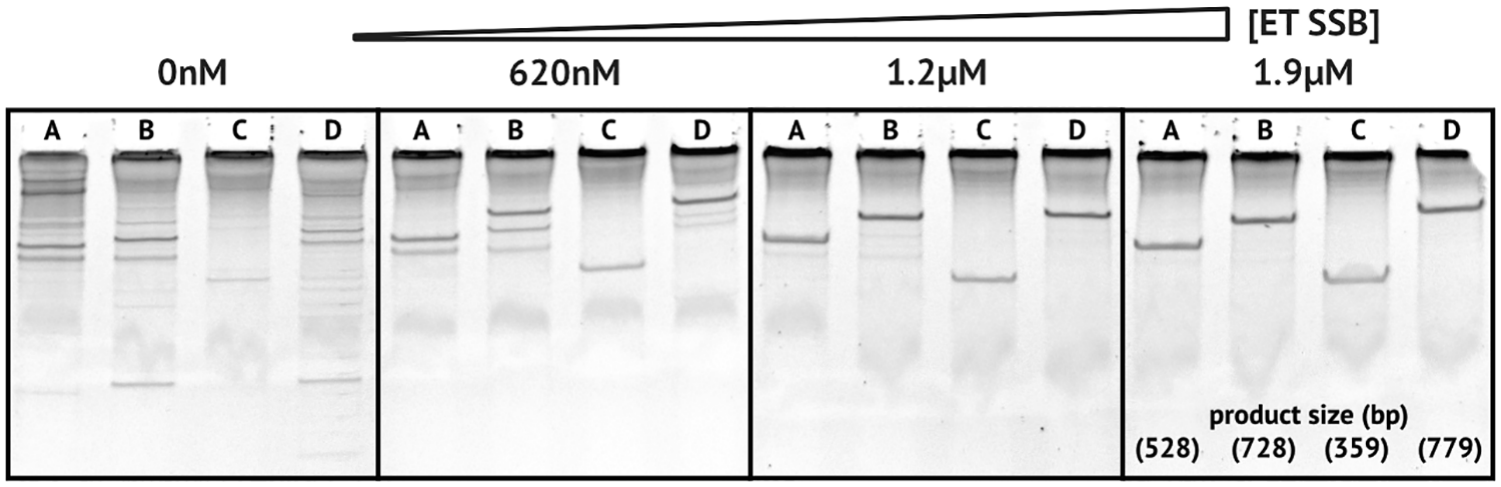
ET SSB reduces off-target activity with *Tt*Ago. Sets of two guides targeting different locations on ΦX174 Virion DNA over increasing concentration of ET SSB. Reactions were carried out at 74 °C for 5 hr and products were analyzed on Novex 20% polyacrylamide TBE gels stained with 3X GelRed in water. Off target cutting is reduced with higher concentrations of ET SSB. (**A**) 528 nt product using ΦX174 guides 3 and 5; (**B**) 728 nt product using ΦX174 guides 3 and 6; (**C**) 359 nt product using ΦX174 guides 1 and 4; (**D**) 779 nt product using ΦX174 guides 2 and 6.

We hypothesize that these critical mismatches in conjunction with the ET SSB may promote mismatched target release prior to the formation of a cleavage competent conformation. Further base pairing beyond the seed region brings the argonaute into a conformation which allows for endonuclease activity [41, 42]. The ability of ET SSB to focus specificity onto a very specific region of the guide could allow for *Tt*Ago and other pAgos to be used in applications, such as recently presented by Lapinaite et al. [40], where binding with a high degree of single-base discrimination is required. As target substrates become larger, the chance for off-target activity due to the presence of similar sequences becomes more likely. We observed that ET SSB also improved the specificity of *Tt*Ago when used to cut specific segments from ΦX174 Virion DNA (Fig 8).

### Similar activity is observed using other thermostable SSB proteins

**Fig 9:**
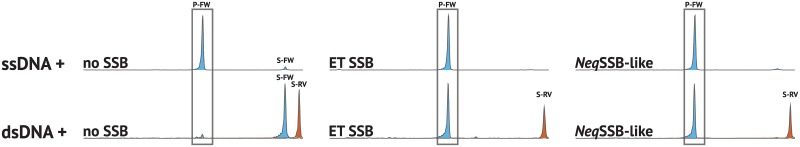
*Neq*SSB-like protein provides similar enhancements to *Tt*Ago activity as ET SSB. CE traces showing the ability of *Tt*Ago to cut linear dsDNA substrates only in the presence of a thermostable SSB protein such as ET SSB or *Neq*SSB-like protein. The product (P-FW) is boxed for easier identification. The blue trace is from the 5′-FAM of the forward strand of the substrate (S-FW), and the red trace is from the 3′-TAMRA of the complement strand of the substrate (S-RV). Reactions were carried out using optimal-length 16 nt guides at 73 °C for 8 hr to demonstrate the low activity of *Tt*Ago in the absence of SSB on dsDNA substrate. Only in reactions containing SSB could *Tt*Ago cut dsDNA substrate. From left to right, reactions contained: no SSB; 1.2 *μ*M ET SSB; and 420 nM *Neq*SSB-like protein.

To verify the general principles of adding ET SSB wtih pAgo to enhance activity, another thermostable SSB—the SSB protein from *Nanoarchaeum equitans* (*Neq*SSB-like protein)—was cloned, expressed, and purified from *E. coli*. *Neq*SSB-like protein was selected as an alternative to ET SSB for its high thermostability. Additionally, *Neq*SSB-like protein is capable of binding ssDNA, RNA, and dsDNA [63]. Given this unique property, we hypothesized a binding protein such as this could also provide a similar activity-enhancing effect for pAgos. The addition of *Neq*SSB-like protein also allowed *Tt*Ago to cleave linear dsDNA, as observed with ET SSB (Fig 9). In the absence of SSB no activity was observed on dsDNA substrates. It should be noted, as shown in Figs 2 and 6, that SSB also increased the rate of activity on ssDNA substrates in addition to allowing activity on dsDNA substrates. In order to demonstrate that the presence of SSB was enabling activity on otherwise inaccessible double-stranded substrates, as opposed to simply increasing the rate of activity on said substrates, the reactions were incubated at 73 °C for 8 hr using 16 nt 5′-phosphorylated guides. Previous experiments in this study showed that guides 16 nt in length were the most active with *Tt*Ago. Using this optimal guide length in conjunction with the longer incubation time ensured that native *Tt*Ago without ET SSB or *Neq*SSB-like protein added would exhibit some activity on dsDNA substrates if possible.

While the same end result was achieved using both ET SSB and *Neq*SSB-like protein (Fig 9), the two SSBs were not equivalent in their function with *Tt*Ago. Both SSBs had a critical concentration beyond which inhibition of *Tt*Ago activity was observed likely through competition with *Tt*Ago for binding (see Fig 6 at ET SSB concentration of 1.5 *μ*M), but *Neq*SSB-like protein had a much narrower functional window and needed to be used at a much lower concentration. This could be due to the more prominent ability of *Neq*SSB-like protein to bind to dsDNA as well as ssDNA (Supporting information S7 Fig). Perhaps binding of the dsDNA, which is a slightly less preferred substrate to ssDNA [63], in fact further prevents *Tt*Ago from acting on the dsDNA substrate by making it inaccessible.

### Unique sticky ends generated by *Tt*Ago dual-guide reactions

**Fig 10:**
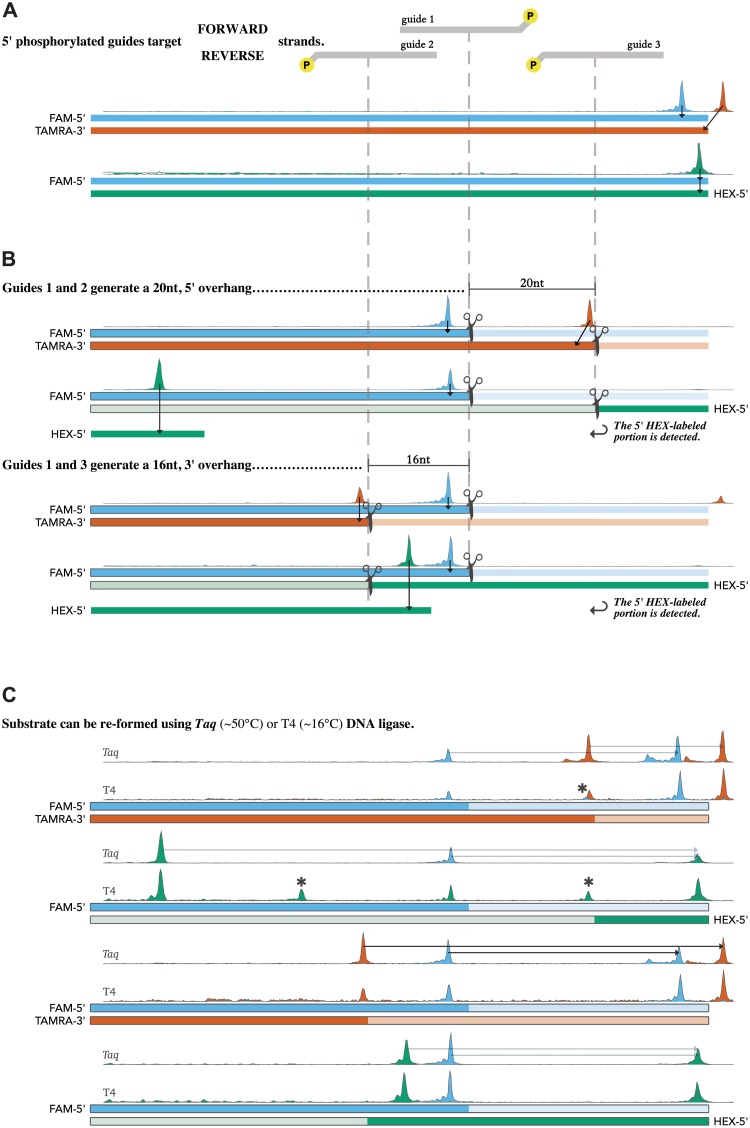
*Tt*Ago as a programmable restriction endonuclease. Argonaute endonuclease reactions were performed in *Taq* DNA Ligase Reaction Buffer at 74 °C for 2 hr. Upon completion, the disappearance of substrate was monitored by CE, and the reactions were subsequently split and treated with either *Taq* or T4 DNA ligase supplemented with NAD^+^ or ATP, respectively. Full-length product was then re-annealed, ligated, and detected by CE—fluorescence peak data is shown above each substrate/product diagram. (**A**) The TAMRA dye is attached to the synthetic oligo via a modified thymidine; as such, the two targets differ in sequence by one base—a T in the 3′ terminal position of the TAMRA-labeled oligo becomes a G in the HEX-labeled oligo. This causes a slight difference in capillary migration time for the two FAM-labeled products. (**B**) Fluorophore hydrophobicity also affects product migration in the capillary; therefore, the peaks displayed correlate with the size as determined by a standard, which slightly differs from the actual length of the product. (**C**) T4 DNA ligase is able to generate a broader palate of ligation products than Taq DNA ligase due to increased functionality at lower temperatures and the ability to perform blunt end ligation. Peaks marked with (*) arise from ligation events involving excess 5′-phosphorylated DNA guide.

To demonstrate the usability of *Tt*Ago with ET SSB as a molecular tool, the combination was used as a programmable restriction endonuclease to generate double-strand breaks in a randomized dsDNA substrate with unique 20 nt 5′-overhangs, as well as unique 16 nt and 6 nt 3′-overhangs (Fig 10A–10B). Enghiad and Zhao demonstrated the use of *Pf* Ago as a programmable restriction endonuclease at temperatures ranging from 87–98 °C [31]. In a similar manner *Tt*Ago is able to generate longer overhangs than currently possible with traditional restriction endonuclease s, however, in the presence of ET SSB this can be done at temperatures which do not cause full duplex melting as with *Pf* Ago. Following the successful creation of these overhangs using *Tt*Ago and ET SSB, *Taq* and T4 DNA ligase were used separately to regenerate full length product from the annealed sticky ends to regenerate a full length fluorescent dye-labeled product that could also be quantified by CE analysis (Fig 10C). The longer overhangs, 20 nt 5′- and 16 nt 3′-overhangs were successfully ligated by *Taq* DNA ligase at elevated temperature (50 °C, Fig 10C), whereas the 6 nt overhang was not long enough to be ligated by *Taq* DNA ligase at this temperature. The ability to generate unique, variable-length overhangs without specific sequence requirements as imposed by traditional restriction endonucleases enables ligation to be carried out at higher temperatures for increased fidelity, and could be useful for assembly methods that can organize multiple segments with high accuracy. Additionally, this allows for cloning techniques that are not limited to traditional restriction endonuclease sequences. Sequence integration or modification could therefore be achieved at any site within a sequence, e.g. synthetic biology applications or site-specific cassette insertions into fusion-protein constructs. With the ability to act on dsDNA substrates, *Tt*Ago with ET SSB could also be used in depletion strategies for next-generation sequencing, specifically targeting and cutting unwanted or contaminating sequences prior to library preparation [36].

When using *Tt*Ago to generate double-strand breaks, it was observed that a short incubation of the individual guides with *Tt*Ago for 10–15 min at 75 °C improved the efficiency of the reaction. While it has been observed that *Tt*Ago can utilize guides without preference for specific bases in key positions—for example at or in close proximity to the 5′-phosphorylated end—DNA guides co-purifying with *Tt*Ago have been reported to have a strong preference for deoxycytidine in the first position and deoxyadenosine in the second position [3, 26]. As such we hypothesize that while *Tt*Ago is able to utilize guides of all varieties, loading may be far more efficient with certain preferred sequences, and therefore an improvement in double-strand break generation following pre-incubation with guides could be due to the elimination of competition between guides for loading, allowing more equal targeting of both strands.

### Thermostable SSB did not improve *Ng*Ago activity

A recent (now retracted) study [32] garnered significant attention to the exciting possibility of using *Ng*Ago for DNA-guided dsDNA nicking at physiological temperatures and accordingly a novel gene editing tool. While these claims have not been borne out by subsequent investigation, it is now likely the case that *Ng*Ago can act on RNA [35]. Given the unique temperature profile and potential of using *Ng*Ago, we investigated whether ET SSB or *Neq*SSB-like protein could improve or impart enhanced activity to *Ng*Ago, however we did not observe any DNA cleavage with or without the addition of thermostable SSBs. While this result did not add to this work pertaining to the use of accessory factors with pAgos targeting DNA substrates, we did detect some specific DNA-guided RNA endonuclease activity. Given the recent attention of the scientific community to *Ng*Ago, further detail is given in the Supporting information S1 Appendix.

## Conclusion

There has been considerable interest in pAgos—beyond aspirations for their application in gene editing, the endonuclease activity coupled with a sequence specific guidance system, gives them great potential to be useful molecular tools for a variety of different nucleic acid manipulations and applications. The lack of a PAM sequence, as required by Cas enzymes, and the ability to use inexpensive and easily-synthesized DNA guides make pAgos an attractive option for target enrichment or depletion strategies and high-throughput guided-nuclease applications, provided that the catalytic activity is high and operates on dsDNA substrates. The use of *Tt*Ago with SSBs or other accessory factors addresses these issues and improves pAgo function in such applications. Enhanced mismatch discrimination and activity on double-stranded DNA substrates by including ET SSB with *Tt*Ago enables potential use of pAgos in these strategies with single-base resolution. For *in vitro* usage, the addition of accessory proteins affecting nucleic acid secondary structure, such as helicases or SSBs, can significantly improve pAgo activity on otherwise inaccessible substrates, notably dsDNA and high GC-content sequences which could exhibit considerable secondary structure or inefficient product release. As a general method, the use of accessory factors such as helicase or SSBs greatly improves pAgos as an *in vitro* molecular tool, and further studies will focus on combining this unique functionality with a variety of downstream applications.

## Materials and methods

### Materials

The pMiniT™ 2.0 vector; pUC19 vector; pBR322 vector; ΦX174 Virion, RFI, and RFII DNA; Extreme-Thermostable Single-Stranded DNA Binding Protein (ET SSB); T4 Gene 32 Protein (gp32); Proteinase K; SspI-HF^®^; Nt.BspQI; ThermoPol^®^ Reaction Buffer (20 mM Tris(hydroxymethyl)aminomethane hydrochloride (Tris-HCl) pH 8.8 @ 25 °C, 10 mM potassium chloride, 10 mM ammonium sulfate, 2 mM magnesium sulfate, and 0.1% Triton X-100); *Taq* DNA Ligase Reaction Buffer (20 mM Tris-HCl pH 7.6 @ 25 °C, 25 mM potassium acetate, 10 mM magnesium acetate, 1 mM NAD^+^, 10 mM dithiothreitol (DTT), and 0.1% Triton X-100); NEBuffer™ 3.1; NEBuffer™ 4; CutSmart^®^ Buffer; Diluent E; and Quick-Load Purple 2-Log and Low Molecular Weight (LMW) DNA Ladders were obtained from New England Biolabs, Inc. (NEB; Ipswich, Massachusetts, USA). Novex™ 20% polyacrylamide TBE Gels were obtained from Thermo Fisher Scientific, Inc. (Waltham, Massachusetts, USA). GelRed™ (Biotium, Inc., Fremont, California, USA) was obtained from VWR (Radnor, Pennsylvania, USA).

Synthetic DNA and RNA oligonucleotides were purchased from Integrated DNA Technologies (Coralville, Iowa, USA). Guide oligonucleotides were phosphorylated using NEB^®^ T4 Polynucleotide Kinase (PNK) according to the provided product protocol for non-radioactive phosphorylation. PNK was thermally denatured by incubation at 65 °C for 20 min following the phosphorylation reaction, and phosphorylated guides were used directly. Single and double-stranded DNA and RNA substrates were modified with a 5′-FAM (carboxyfluorescein) on the forward strand. Double-stranded DNA substrates were also modified with either a 3′-TAMRA (carboxytetramethylrhodamine) or 5′-HEX (hexachlorofluorescein) label on the reverse strand. Sequence information for guides and fluorescent dye-labeled substrates can be found in the Supporting information S1 and S2 Tables.

*Pf* Ago and UvrD-like helicases from multiple organisms were generously provided through in-house collaborations at NEB and were used as provided without further modification or supplementation.

### Expression and purification of *Tt*Ago

*Tt*Ago was obtained as a synthetic, codon-optimized sequence with amino-terminal hexahistidine tag from GenScript in plasmid pET28c and transformed into NEB Turbo competent *E. coli* for cloning and propagation, and T7 Express lysY/Iq competent *E. coli* for expression. Large scale cultures were grown to OD_600_ 0.6 at 37 °C, induced with 0.5 mM IPTG, expressed overnight (14 hr) at 16 °C, and purified by immobilized metal ion affinity chromatography (IMAC) using gravity flow with Nickel NTA Agarose Beads (Gold Biotechnology, St. Louis, Missouri), or fast protein liquid chromatography (FPLC) using a 5mL HisTrap FF column on an ÄKTA FPLC (GE Healthcare Life Sciences)—both methods were effective at producing functional, high-purity argonaute. Purified fractions were dialyzed into a high-salt storage buffer of 10 mM Tris-HCl, pH 7.5 @ 25 °C; 0.5 M sodium chloride; 2 mM magnesium chloride; 1 mM DTT; 0.1 mM ethylenediaminetetraacetic acid (EDTA); 0.1% Triton X-100; and 50% glycerol and stored at -20 °C.

Endonuclease activity on supercoiled plasmids was verified according to previously published methods [23] (see Supporting information S1 Fig). *Tt*Ago was used in conjunction with five different pairs of guides to generate a double-strand break in a PCR-amplified sequence ligated into the pMiniT™ 2.0 vector (NEB). *Tt*Ago was mixed with guides and substrate in a 25:5:1 molar ratio, respectively, incubated for 16 hr at 75 °C, halted by the addition of proteinase K, and analyzed by agarose gel electrophoresis. Subsequent reactions to determine optimal buffer conditions were carried out using supercoiled pUC19 plasmid as a substrate and guides designed to target specific regions within the multiple cloning site (see Supporting information S2–S4 Figs).

### Expression and purification of *Neq*SSB-like protein

Thermostable SSB from *Nanoarchaeum equitans* (*Neq*SSB-like protein) was obtained as a synthetic, codon-optimized sequence with amino-terminal hexahistidine tag from GenScript in plasmid pET29a(+) and transformed into NEB Turbo competent *E. coli* for cloning and propagation, and T7 Express Competent *E. coli* for expression. Large scale cultures were grown to OD_600_ 0.6 at 30 °C, induced with 0.5 mM IPTG, expressed at 16 °C overnight (14 hr), and purified by IMAC FPLC using a 5mL HisTrap FF column on an ÄKTA FPLC (GE Healthcare Life Sciences). Purified fractions were dialyzed into a storage buffer of 20 mM Tris-HCl, pH 7.5 @ 25 °C; 200 mM sodium chloride; 0.5 mM DTT; 1 mM EDTA; and 50% glycerol and stored at -20 °C.

### Variables affecting *Tt*Ago activity

Following expression and purification of *Tt*Ago, endonuclease activity was verified according to the methods described in [23]. Briefly, sets of two guides targeting complementary strands were used to generate linearized plasmid from supercoiled pMiniT™ 2.0 substrate (Supporting information S1 Fig). Additionally, several buffer conditions were screened to identify optimal conditions for *Tt*Ago activity. Optimal pH was determined to be approximately 8.8 with a functional range from >7.5 to <9.5 (Supporting information S2 Fig), and optimal temperature was determined to be approximately 73–74 °C with a functional range from >65 to <75 °C (Supporting information S3 Fig). Salt concentrations, specifically chloride salts, above 70 mM greatly inhibited activity (Supporting information S4 Fig). The use of potassium chloride in place of sodium chloride was favorable, and under these conditions reliable activity was observed with chloride, sulfate, or acetate salts of magnesium(II). Manganese(II) chloride was also functional. The addition of 2–10% glycerol to the reaction buffer was well tolerated and had minimal effect on *Tt*Ago activity. ThermoPol^®^ Reaction Buffer was chosen as an optimal buffer for all subsequent reactions as it was within the functional parameters listed above and provided consistent results and reaction conditions amenable to experiments involving other proteins in conjunction with *Tt*Ago.

### Single-guide argonaute reactions

Substrate oligonucleotides were synthesized by IDT and contained either a FAM, TAMRA, or HEX label. Guides were synthesized by IDT and phosphorylated using T4 PNK according to the instructions for non-radioactive phosphorylation provided with the product. All assays utilized 21 nt guides unless specified otherwise. *Tt*Ago (1 *μ*M), guide (5 *μ*M), and substrate (30 nM) were mixed in a 2:1:1 molar ratio. The reactions were carried out at 73 °C in ThermoPol^®^ Reaction Buffer. Reactions were halted by rapid cooling followed by the addition of 1 *μ*L of Proteinase K and incubation at 45 °C for 1 hr. Reactions were diluted to a substrate concentration of 4 nM prior to CE analysis, as previously described in [64]. CE benefits from high sensitivity, short time-to-result, and an inherent capacity for high-throughput assay design, which allowed for the rapid, reliable screening of multiple factors on a variety of substrates with replicates. CE analysis was performed on an Applied Biosystems 3730xl DNA Analyzer, 36 cm capillary array with 5 s injection time. Peaks were sized according to the GeneScan™ 120 LIZ™ dye Size Standard (Thermo Fisher Scientific).

Plasmid substrate-based assays were performed at 73 °C in 20 mM Bis-Tris propane, pH 8.8, 40 mM sodium chloride, 2 mM magnesium acetate unless otherwise specified, followed by Proteinase K treatment as above. Products of the reaction were visualized by agarose gel electrophoresis with 2-Log and LMW DNA standards (NEB) and stained with 1X GelRed™.

### Dual-guide argonaute reactions

To generate double-strand breaks with various types of topology, pairs of guides targeting opposite strands were selected and loaded individually into *Tt*Ago by mixing in a 5:1 excess (guide:*Tt*Ago) and incubating at 70 °C for 20 min. All assays utilized 21 nt guides unless specified otherwise. The separately pre-loaded *Tt*Agos were then combined with each other and substrate oligonucleotide in a 5:1:1 molar ratio (guide:*Tt*Ago:target). The reactions were carried out at 74 °C in *Taq* DNA Ligase Reaction Buffer. Reactions were halted by rapid cooling followed by the addition of 1 *μ*L of Proteinase K and incubation at 45 °C for 1 hr. Reactions were diluted to a substrate concentration of 4 nM prior to CE analysis, as previously described in [64].

### Data analysis and figure preparation

Data handling and analysis was performed with Microsoft Excel (Microsoft Corporation, Redmond, Washington, USA) and GraphPad Prism (GraphPad Software, Inc., La Jolla, California, USA). Figures were prepared using Affinity Designer and Affinity Photo (Serif, Ltd., Nottingham, UK). Gels were imaged and processed using an AlphaImager HP system (ProteinSimple, San Jose, California). Quantitative analysis of CE data was performed using PeakScanner Software v1.0 (Thermo Fisher Scientific, Inc., Waltham, Massachusetts, USA) and fragment analysis software developed for in-house use at New England Biolabs [64].

## Supporting information

S1 Fig *Tt*Ago cuts supercoiled plasmid. NEB^®^ pMiniT™ 2.0 containing a PCR amplified sequence is linearized by *Tt*Ago using five sets of 21 nt 5′-phosphorylated guides targeting complementary strands according to the methods described in [23]. Lanes 1–5 contain reactions corresponding to each set of forward and reverse guides incubated at 75 °C for 16 hr; Lane 6 contains plasmid controls, nicked (N) with Nt.BspQI, linearized (L) with SspI-HF, and supercoiled (S).
(PNG)

S2 Fig Effect of pH on *Tt*Ago activity. Supercoiled pUC19 plasmid is linearized by *Tt*Ago using a set of 21 nt 5′-phosphorylated guides targeting complementary strands at varying pH. *Tt*Ago shows no activity in the absence of a 5′-phosphate on the guides. The product band (linearized pUC19) is identified by an arrow. Plasmid controls (C) consist of pUC19 nicked (N) with Nt.BspQI, linearized (L) with EcoRI, and supercoiled (S). The marker (M) is NEB^®^ Quick-Load Purple 2-Log DNA Ladder.
(PNG)

S3 Fig Effect of salt and temperature on *Tt*Ago activity. Supercoiled pUC19 plasmid is linearized by *Tt*Ago using a set of 21 nt 5′-phosphorylated guides targeting complementary strands at a varying sodium chloride concentrations and across a gradient of temperatures. Chloride concentration was carefully controlled by using a reaction buffer consisting of 20 mM Bis-tris propane, adjusted to pH 8.8 with acetic acid, 2 mM magnesium sulfate, and varying amounts of sodium chloride spiked in. A minimal amount of sodium chloride is carried over from the *Tt*Ago storage buffer (∼1 mM). Activity drops off sharply above 50 mM salt, and the most consistent activity was observed at 73 °C. The product band (linearized pUC19) is identified by arrows. Plasmid controls (C) consist of pUC19 nicked (N) with Nt.BspQI, linearized (L) with EcoRI, and supercoiled (S). The marker (M) is NEB Quick-Load Purple 2-Log DNA Ladder.
(PNG)

S4 Fig Effect of salt on *Tt*Ago activity in the presence of ET SSB. Salt sensitivity of *Tt*Ago in the presence of ET SSB determined by activity on fluorescent dye-labeled CE DNA substrates of varying GC content. A) ssDNA, 30%GC; B) ssDNA, 50%GC; C) ssDNA, 70%GC; D) dsDNA, 50%GC (see S2 Table for sequences). Percent completion was determined as the ratio of product to substrate identified by CE following a 1 hr reaction at 73 °C.
(PDF)

S5 Fig *Tt*Ago with UvrD-like helicases from various other thermophilic bacterial and archaeal organisms. (A, D) Pya (*Pyrococcus yayanosii*); Tko (*Thermococcus kodakarensis*), the (*) signifies the sequence differs from the wildtype; Tth (*Thermus thermophilus*), (10X) refers to the helicase concentration compared to the following lane; Tte (*Thermoanaerobacter tengcongensis*). Reactions were performed in NEBuffer™ 4 with 1 mM ATP (A) or without ATP (D). Amount of helicase varied from 10–50ng per reaction. Guides were pre-loaded by incubation at 75 °C for 20 min followed by incubation for reaction at 73 °C for 4 hr. Reactions were halted by the addition of Proteinase K, incubated at 45 °C for 30 min. Guides were designed to target the BamHI site in supercoiled pUC19 substrate. Product was observed by the appearance of linearized pUC19 (A), but was not observed in the absence of ATP (D). For comparison ET SSB was also added under the same reaction conditions. Increased activity was observed with ET SSB which also showed some off-target cleavage generating a second product band. Increased activity was observed with the *Tth*UvrD and perhaps some activity with the other bacterial *Tte*UvrD, but not with the archaeal UvrD-like proteins. To verify that the cleavage products observed were not due to contamination in the *Tth*UvrD or ET SSB preps, *Tth*UvrD only (B) and ET SSB only (C) controls were carried out on supercoiled and linearized pBR322 plasmid at 73 °C for 8 hr. No degradation of either substrate was observed. Plasmid controls (ctrls) are nicked (N) with Nt.BspQI, linearized (L) with EcoRI, and supercoiled (S). The marker (M) is NEB Quick-Load Purple 2-Log DNA Ladder in (A, D), and NEB Fast DNA Ladder in (B, C).
(JPG)

S6 Fig Non-specific activity of *Pf* Ago on double-stranded DNA substrates is increased in the presence of ET SSB. B) With an exposed 5′-phosphate present on the substrate, increasing guide concentration only marginally delays non-specific degradation of the substrate. C) Even in the absence of a 5′-phosphate and with excess guide, non-specific degradation of the substrate occurs in the presence of ET SSB.
(PDF)

S7 Fig SSB binding activity. SSB binding activity for gp32, ET SSB, and *Neq*SSB-like protein observed by gel shift on 0.8% agarose TBE gel electrophoresis with ssDNA (ΦX174 virion) and relaxed, circular dsDNA (ΦX174 RFII).
(PNG)

S8 Fig Individual point mutations of the *Tt*Ago catalytic tetrad eliminate activity. Point mutations D478A, E512A, D546A, and D660A were introduced separately using the Q5^®^Mutagenesis Kit (New England Biolabs) following the instructions provided with the kit. These four residues are known to be involved in the RNase H-type active site of *Tt*Ago [20]. Mutants were prepared and purified according to the same expression and purification procedures provided in the Materials and methods section. Mutation of the active site eliminated endonuclease activity indicating that when taken in conjunction with the other controls and data provided in this manuscript, the activity observed with the non-mutated sequence can be genuinely accredited to *Tt*Ago and not a contaminating nuclease. Reactions were carried out using guide set 5 as in Supporting information S1 Fig.
(PNG)

S1 Table Sequence and GC content information for guides. Left→Right, 5′→3′; cleavage site between bases 10 and 11.
(PDF)

S2 Table Sequence and GC content information for CE substrates. (PDF)

S1 Appendix Methods and discussion pertaining to *Ng*Ago and ET SSB. ET SSB did not improve *Ng*Ago activity as a DNA-guided DNA or RNA endonuclease.
(PDF)

## References

[1] Koonin EV. Evolution of RNA- and DNA-guided antivirus defense systems in prokaryotes and eukaryotes: common ancestry vs convergence. Biol Direct. 2017;12(1):561 10.1186/s13062-017-0177-2 PMC5303251 28187792

[2] Zhu L, Jiang H, Sheong FK, Cui X, Wang Y, Gao X, et al Understanding the core of RNA interference: The dynamic aspects of Argonaute-mediated processes. Prog Biophys Mol Biol. 2017;128:39–46. 10.1016/j.pbiomolbio.2016.09.008 27697475

[3] Swarts DC, Makarova K, Wang Y, Nakanishi K, Ketting RF, Koonin EV, et al The evolutionary journey of Argonaute proteins. Nat Struct Mol Biol. 2014;21(9):743–753. 10.1038/nsmb.2879 25192263 PMC4691850

[4] Makarova KS, Wolf YI, Koonin EV. Comparative genomics of defense systems in archaea and bacteria. Nucleic Acids Res. 2013;41(8):4360–4377. 10.1093/nar/gkt157 23470997 PMC3632139

[5] Koonin EV, Makarova KS, Wolf YI. Evolutionary Genomics of Defense Systems in Archaea and Bacteria. Annu Rev Microbiol. 2017;71(1):233–261. 10.1146/annurev-micro-090816-093830 28657885 PMC5898197

[6] Hegge JW, Swarts DC, van der Oost J. Prokaryotic Argonaute proteins: novel genome-editing tools? Nat Rev Microbiol. 2017;434:356. 10.1038/nrmicro.2017.73 28736447

[7] Kwak PB, Tomari Y. The N domain of Argonaute drives duplex unwinding during RISC assembly. Nat Struct Mol Biol. 2012;19(2):145–151. 10.1038/nsmb.2232 22233755

[8] Sheu-Gruttadauria J, MacRae IJ. Structural Foundations of RNA Silencing by Argonaute. J Mol Biol. 2017;429(17):2619–2639. 10.1016/j.jmb.2017.07.018 28757069 PMC5576611

[9] Yan KS, Yan S, Farooq A, Han A, Zeng L, Zhou MM. Structure and conserved RNA binding of the PAZ domain. Nature. 2003;426(6965):468–474. 10.1038/nature02129 14615802

[10] Lingel A, Simon B, Izaurralde E, Sattler M. Nucleic acid 3´-end recognition by the Argonaute2 PAZ domain. Nat Struct Mol Biol. 2004;11(6):576–577. 10.1038/nsmb777 15156196

[11] Ma JB, Yuan YR, Meister G, Pei Y, Tuschl T, Patel DJ. Structural basis for 5´-end-specific recognition of guide RNA by the A. fulgidus Piwi protein. Nature. 2005;434(7033):666–670. 10.1038/nature03514 15800629 PMC4694588

[12] Parker JS, Roe SM, Barford D. Structural insights into mRNA recognition from a PIWI domain–siRNA guide complex. Nature. 2005;434(7033):663–666. 10.1038/nature03462 15800628 PMC2938470

[13] Parker JS, Roe SM, Barford D. Crystal structure of a PIWI protein suggests mechanisms for siRNA recognition and slicer activity. EMBO J. 2004;23(24):4727–4737. 10.1038/sj.emboj.7600488 15565169 PMC535097

[14] Song JJ. Crystal Structure of Argonaute and Its Implications for RISC Slicer Activity. Science. 2004;305(5689):1434–1437. 10.1126/science.1102514 15284453

[15] Azlan A, Dzaki N, Azzam G. Argonaute: The executor of small RNA function. J Genet Genomics. 2016;43(8):481–494. 10.1016/j.jgg.2016.06.002 27569398

[16] Yuan YR, Pei Y, Ma JB, Kuryavyi V, Zhadina M, Meister G, et al Crystal Structure of A. aeolicus Argonaute, a Site-Specific DNA-Guided Endoribonuclease, Provides Insights into RISC-Mediated mRNA Cleavage. Mol Cell. 2005;19(3):405–419. 10.1016/j.molcel.2005.07.011 16061186 PMC4689305

[17] Wang Y, Juranek S, Li H, Sheng G, Tuschl T, Patel DJ. Structure of an argonaute silencing complex with a seed-containing guide DNA and target RNA duplex. Nature. 2008;456(7224):921–926. 10.1038/nature07666 19092929 PMC2765400

[18] Wang Y, Sheng G, Juranek S, Tuschl T, Patel DJ. Structure of the guide-strand-containing argonaute silencing complex. Nature. 2008;456(7219):209–213. 10.1038/nature07315 18754009 PMC4689319

[19] Wang Y, Juranek S, Li H, Sheng G, Wardle GS, Tuschl T, et al Nucleation, propagation and cleavage of target RNAs in Ago silencing complexes. Nature. 2009;461(7265):754–761. 10.1038/nature08434 19812667 PMC2880917

[20] Sheng G, Zhao H, Wang J, Rao Y, Tian W, Swarts DC, et al Structure-based cleavage mechanism of Thermus thermophilus Argonaute DNA guide strand-mediated DNA target cleavage. Proc Natl Acad Sci. 2014;111(2):652–657. 10.1073/pnas.1321032111 24374628 PMC3896195

[21] Willkomm S, Oellig CA, Zander A, Restle T, Keegan R, Grohmann D, et al Structural and mechanistic insights into an archaeal DNA-guided Argonaute protein. Nat Microbiol. 2017;2:17035 10.1038/nmicrobiol.2017.35 28319084

[22] Olovnikov I, Chan K, Sachidanandam R, Newman DK, Aravin AA. Bacterial Argonaute Samples the Transcriptome to Identify Foreign DNA. Mol Cell. 2013;51(5):594–605. 10.1016/j.molcel.2013.08.014 24034694 PMC3809076

[23] Swarts DC, Jore MM, Westra ER, Zhu Y, Janssen JH, Snijders AP, et al DNA-guided DNA interference by a prokaryotic Argonaute. Nature. 2014;507(7491):258–261. 10.1038/nature12971 24531762 PMC4697943

[24] Swarts DC, Koehorst JJ, Westra ER, Schaap PJ, van der Oost J. Effects of Argonaute on Gene Expression in Thermus thermophilus. PLoS One. 2015;10(4):e0124880 10.1371/journal.pone.0124880 25902012 PMC4406477

[25] Swarts DC, Hegge JW, Hinojo I, Shiimori M, Ellis MA, Dumrongkulraksa J, et al Argonaute of the archaeon Pyrococcus furiosus is a DNA-guided nuclease that targets cognate DNA. Nucleic Acids Res. 2015;43(10):5120–5129. 10.1093/nar/gkv415 25925567 PMC4446448

[26] Swarts DC, Szczepaniak M, Sheng G, Chandradoss SD, Zhu Y, Timmers EM, et al Autonomous Generation and Loading of DNA Guides by Bacterial Argonaute. Mol Cell. 2017;65(6):985–998.e6. 10.1016/j.molcel.2017.01.033 28262506 PMC5779613

[27] Zander A, Willkomm S, Ofer S, van Wolferen M, Egert L, Buchmeier S, et al Guide-independent DNA cleavage by archaeal Argonaute from Methanocaldococcus jannaschii. Nat Microbiol. 2017;2:17034 10.1038/nmicrobiol.2017.34 28319081 PMC7616673

[28] Meister G, Landthaler M, Peters L, Chen PY, Urlaub H, Lührmann R, et al Identification of Novel Argonaute-Associated Proteins. Curr Biol. 2005;15(23):2149–2155. 10.1016/j.cub.2005.10.048 16289642

[29] Meister G. Argonaute proteins: functional insights and emerging roles. Nat Rev Genet. 2013;14(7):447–459. 10.1038/nrg3462 23732335

[30] Willkomm S, Zander A, Gust A, Grohmann D. A Prokaryotic Twist on Argonaute Function. Life. 2015;5(1):538–553. 10.3390/life5010538 25692904 PMC4390867

[31] Enghiad B, Zhao H. Programmable DNA-Guided Artificial Restriction Enzymes. ACS Synth Biol. 2017;6(5):752–757. 10.1021/acssynbio.6b00324 28165224

[32] Gao F, Shen XZ, Jiang F, Wu Y, Han C. DNA-guided genome editing using the Natronobacterium gregoryi Argonaute. Nat Biotechnol. 2016;34(7):768–773. 10.1038/nbt.3547 27136078

[33] Lee SH, Turchiano G, Ata H, Nowsheen S, Romito M, Lou Z, et al Failure to detect DNA-guided genome editing using Natronobacterium gregoryi Argonaute. Nat Biotechnol. 2016;35(1):17–18. 10.1038/nbt.3753 27893702 PMC5662444

[34] Javidi-Parsijani P, Niu G, Davis M, Lu P, Atala A, Lu B. No evidence of genome editing activity from Natronobacterium gregoryi Argonaute (NgAgo) in human cells. PLoS One. 2017;12(5):e0177444 10.1371/journal.pone.0177444 28494027 PMC5426773

[35] Sunghyeok Y, Taegeun B, Kyoungmi K, Omer H, Seung Hwan L, Yoon Young K, et al. DNA-dependent RNA cleavage by the Natronobacterium gregoryi Argonaute; 2017.

[36] Gu W, Crawford E, B O, Wilson M, Chow E, Retallack H, et al Depletion of Abundant Sequences by Hybridization (DASH): using Cas9 to remove unwanted high-abundance species in sequencing libraries and molecular counting applications. Genome Biol. 2016;17(1):41 10.1186/s13059-016-0904-5 26944702 PMC4778327

[37] Pardee K, Green AA, Takahashi MK, Braff D, Lambert G, Lee JW, et al Rapid, Low-Cost Detection of Zika Virus Using Programmable Biomolecular Components. Cell. 2016;165(5):1255–1266. 10.1016/j.cell.2016.04.059 27160350

[38] Tsai Y, Greenberg D, Powell J, Hoijer I, Ameur A, Strahl M, et al. Amplification-free, CRISPR-Cas9 Targeted Enrichment and SMRT Sequencing of Repeat-Expansion Disease Causative Genomic Regions; 2017.

[39] Gootenberg J, Abudayyeh O, Lee J, Essletzbichler P, Dy A, Joung J, et al Nucleic acid detection with CRISPR-Cas13a/C2c2. Science. 2017;356(6336):438–442. 10.1126/science.aam9321 28408723 PMC5526198

[40] Lapinaite A, Doudna JA, Cate JHDH. Programmable RNA recognition using a CRISPR-associated Argonaute. Proc Natl Acad Sci USA. 2018;115(13):201717725 10.1073/pnas.1717725115 PMC5879674 29531059

[41] Wee LM, Flores-Jasso CF, Salomon WE, Zamore PD. Argonaute Divides Its RNA Guide into Domains with Distinct Functions and RNA-Binding Properties. Cell. 2012;151(5):1055–1067. 10.1016/j.cell.2012.10.036 23178124 PMC3595543

[42] Klein M, Chandradoss SD, Depken M, Joo C. Why Argonaute is needed to make microRNA target search fast and reliable. Semin Cell Dev Biol. 2017;65:20–28. 10.1016/j.semcdb.2016.05.017 27235676

[43] Lilley DM. DNA opens up–supercoiling and heavy breathing. Trends Genet. 1988;4(4):111–114. 10.1016/0168-9525(88)90099-6 3070859

[44] Jeon JH, Adamcik J, Dietler G, Metzler R. Supercoiling induces denaturation bubbles in circular DNA. Phys Rev Lett. 2010;105(20):208101 10.1103/PhysRevLett.105.208101 21231267

[45] Singleton M, Dillingham M, Wigley D. Structure and Mechanism of Helicases and Nucleic Acid Translocases. Annu Rev Biochem. 2007;76(1):23–50. 10.1146/annurev.biochem.76.052305.115300 17506634

[46] Gilhooly NS, Gwynn EJ, Dillingham MS. Superfamily 1 helicases. Front Biosci (Schol Ed). 2013;5:206–16. 10.2741/S367 23277046

[47] Iftode C, Daniely Y, Borowiec JA. Replication protein A (RPA): the eukaryotic SSB. Crit Rev Biochem Mol Biol. 1999;34(3):141–180. 10.1080/10409239991209255 10473346

[48] Theobald DL, Mitton-Fry RM, Wuttke DS. Nucleic acid recognition by OB-fold proteins. Annu Rev Biophys Biomol Struct. 2003;32:115–133. 10.1146/annurev.biophys.32.110601.142506 12598368 PMC1564333

[49] Zhang J, Zhou R, Inoue J, Mikawa T, Ha T. Single molecule analysis of Thermus thermophilus SSB protein dynamics on single-stranded DNA. Nucleic Acids Res. 2014;42(6):3821–3832. 10.1093/nar/gkt1316 24371279 PMC3973332

[50] Meyer R, reviews LP. The single-stranded DNA-binding protein of Escherichia coli. Microbiol Rev. 1990;. 2087220 10.1128/mr.54.4.342-380.1990 PMC372786

[51] Dabrowski S, Olszewski M, Piatek R, Anna B, Konopa G, Kur J. Identification and characterization of single-stranded-DNA-binding proteins from *Thermus thermophilus* and *Thermus aquaticus*—new arrangement of binding domains. Microbiol Read Engl. 2002;148:3307–15. 10.1099/00221287-148-10-3307 12368464

[52] Richard D, Bell S, White M. Physical and functional interaction of the archaeal single-stranded DNA-binding protein SSB with RNA polymerase. Nucleic Acids Res. 2004;32(3):1065–1074. 10.1093/nar/gkh259 14872062 PMC373395

[53] Dickey TH, Altschuler SE, Wuttke DS. Single-Stranded DNA-Binding Proteins: Multiple Domains for Multiple Functions. Structure. 2013;21(7):1074–1084. 10.1016/j.str.2013.05.013 23823326 PMC3816740

[54] An L, Tang W, Ranalli TA, Kim H, Wytiaz J, Kong H. Characterization of a Thermostable UvrD Helicase and Its Participation in Helicase-dependent Amplification. J Biol Chem. 2005;280(32):28952–28958. 10.1074/jbc.M503096200 15955821 PMC1361353

[55] Tuteja N, Tuteja R. Unraveling DNA helicases. Eur J Biochem. 2004;271(10):1849–1863. 10.1111/j.1432-1033.2004.04094.x 15128295

[56] Hiramatsu Y, Kato R, Kawaguchi Si, Kuramitsu S. Cloning and characterization of the uvrD gene from an extremely thermophilic bacterium, Thermus thermophilus HB8. Gene. 1997;199(1-2):77–82. 10.1016/S0378-1119(97)00349-1 9358042

[57] Oshima RG. Single-stranded DNA binding protein facilitates amplification of genomic sequences by PCR. BioTechniques. 1992;13(2):188 1382464

[58] Rapley R. Enhancing PCR amplification and sequencing using DNA-binding proteins. Mol Biotechnol. 1994;2(3):295–298. 10.1007/BF02745882 7866882

[59] Goldmeyer J, Kong H, Tang W. Development of a Novel One-Tube Isothermal Reverse Transcription Thermophilic Helicase-Dependent Amplification Platform for Rapid RNA Detection. J Mol Diagn. 2007;9(5):639–644. 10.2353/jmoldx.2007.070012 17975029 PMC2049050

[60] Tanner NA, Evans Jr TC. Reducing Template Independent Primer Extension and Threshold Time for Loop Mediated Isothermal Amplification;.

[61] Zhang Y, Tanner NA. Isothermal Amplification of Long, Discrete DNA Fragments Facilitated by Single-Stranded Binding Protein. Sci Rep. 2017;7(1):8497 10.1038/s41598-017-09063-x 28819114 PMC5561150

[62] Zhu L, Jiang H, Sheong FK, Cui X, Gao X, Wang Y, et al A Flexible Domain-Domain Hinge Promotes an Induced-fit Dominant Mechanism for the Loading of Guide-DNA into Argonaute Protein in Thermus thermophilus. J Phys Chem B. 2016;120(10):2709–20. 10.1021/acs.jpcb.5b12426 26908081

[63] Olszewski M, Balsewicz J, Nowak M, Maciejewska N, Cyranka-Czaja A, Zalewska-Piatek B, et al Characterization of a Single-Stranded DNA-Binding-Like Protein from Nanoarchaeum equitans—A Nucleic Acid Binding Protein with Broad Substrate Specificity. PLoS One. 2015;10(5):e0126563 10.1371/journal.pone.0126563 25973760 PMC4431734

[64] Greenough L, Schermerhorn KM, Mazzola L, Bybee J, Rivizzigno D, Cantin E, et al Adapting capillary gel electrophoresis as a sensitive, high-throughput method to accelerate characterization of nucleic acid metabolic enzymes. Nucleic Acids Res. 2016;44(2):e15–e15. 10.1093/nar/gkv899 26365239 PMC4737176

