# Supplementary figures and images for: Single-stranded binding proteins and helicase enhance the activity of prokaryotic argonautes in vitro
Source: PLoS One. 2018 Aug 29;13(8):e0203073. doi: 10.1371/journal.pone.0203073 (PMC6114923; doi:10.1371/journal.pone.0203073)

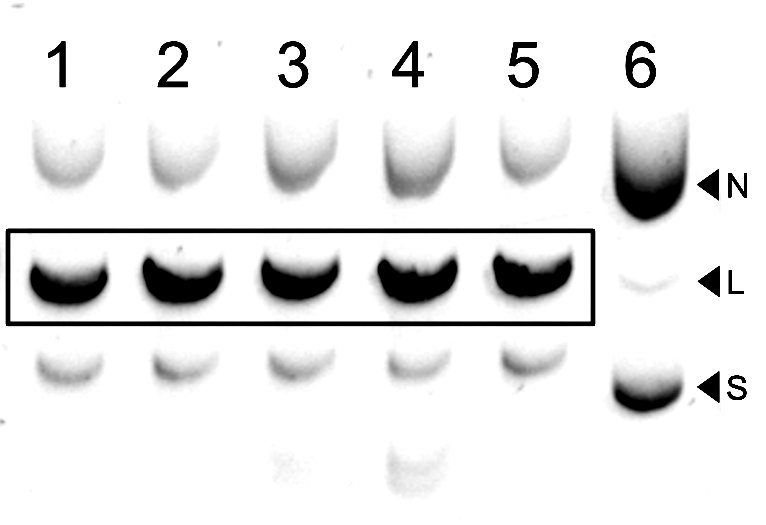

Supplement: S1 Fig — NEB® pMiniT™ 2.0 containing a PCR amplified sequence is linearized by TtAgo using five sets of 21 nt 5′-phosphorylated guides targeting complementary strands according to the methods described in [23]. Lanes 1–5 contain reactions corresponding to each set of forward and reverse guides incubated at 75 °C for 16 hr; Lane 6 contains plasmid controls, nicked (N) with Nt.BspQI, linearized (L) with SspI-HF, and supercoiled (S). (PNG) [file pone.0203073.s001.png]

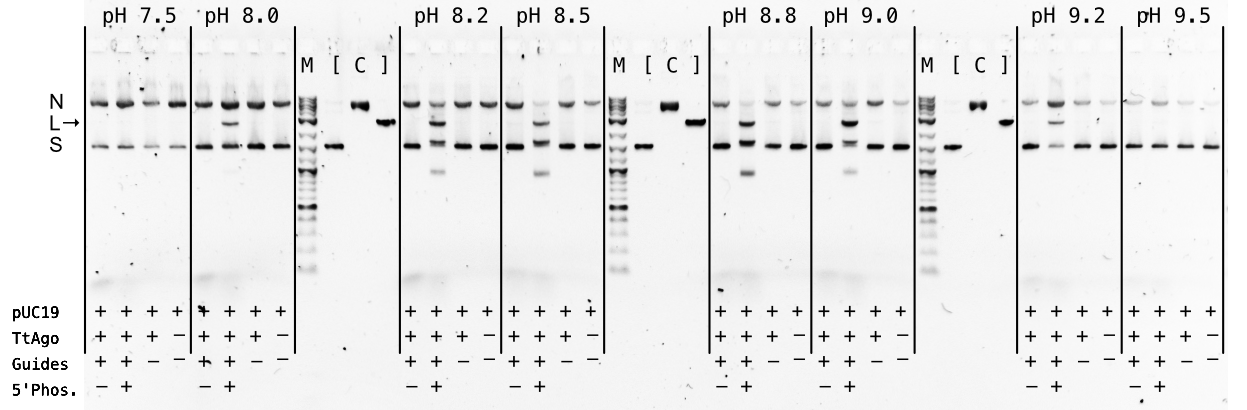

Supplement: S2 Fig — Supercoiled pUC19 plasmid is linearized by TtAgo using a set of 21 nt 5′-phosphorylated guides targeting complementary strands at varying pH. TtAgo shows no activity in the absence of a 5′-phosphate on the guides. The product band (linearized pUC19) is identified by an arrow. Plasmid controls (C) consist of pUC19 nicked (N) with Nt.BspQI, linearized (L) with EcoRI, and supercoiled (S). The marker (M) is NEB® Quick-Load Purple 2-Log DNA Ladder. (PNG) [file pone.0203073.s002.png]

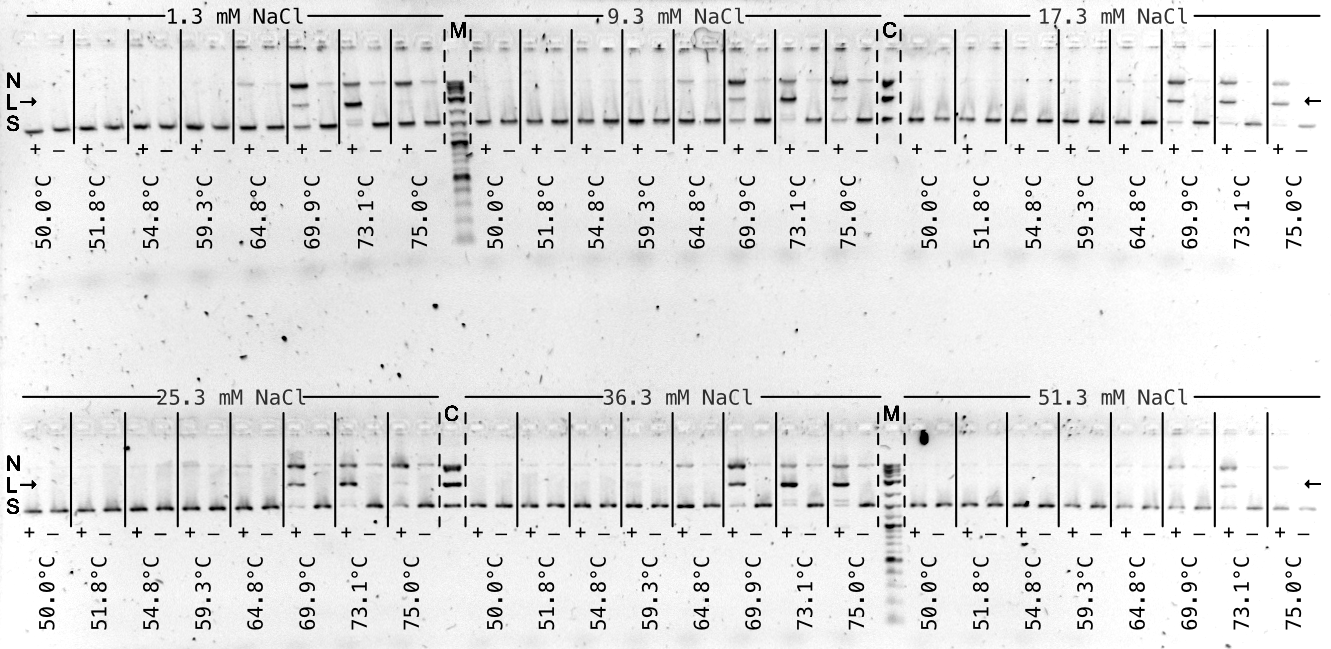

Supplement: S3 Fig — Supercoiled pUC19 plasmid is linearized by TtAgo using a set of 21 nt 5′-phosphorylated guides targeting complementary strands at a varying sodium chloride concentrations and across a gradient of temperatures. Chloride concentration was carefully controlled by using a reaction buffer consisting of 20 mM Bis-tris propane, adjusted to pH 8.8 with acetic acid, 2 mM magnesium sulfate, and varying amounts of sodium chloride spiked in. A minimal amount of sodium chloride is carried over from the TtAgo storage buffer (∼1 mM). Activity drops off sharply above 50 mM salt, and the most consistent activity was observed at 73 °C. The product band (linearized pUC19) is identified by arrows. Plasmid controls (C) consist of pUC19 nicked (N) with Nt.BspQI, linearized (L) with EcoRI, and supercoiled (S). The marker (M) is NEB Quick-Load Purple 2-Log DNA Ladder. (PNG) [file pone.0203073.s003.png]

**A**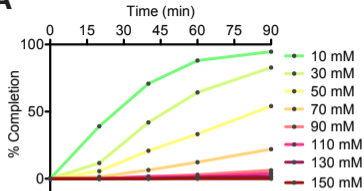**B**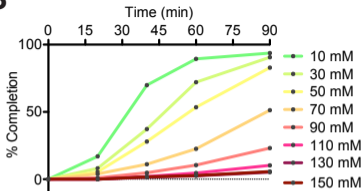**C**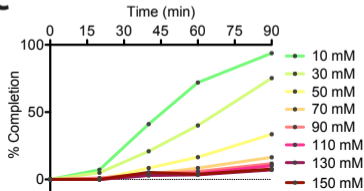**D**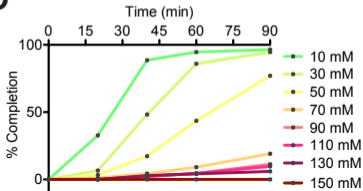

Supplement: S4 Fig — Salt sensitivity of TtAgo in the presence of ET SSB determined by activity on fluorescent dye-labeled CE DNA substrates of varying GC content. A) ssDNA, 30%GC; B) ssDNA, 50%GC; C) ssDNA, 70%GC; D) dsDNA, 50%GC (see S2 Table for sequences). Percent completion was determined as the ratio of product to substrate identified by CE following a 1 hr reaction at 73 °C. (PDF) [file pone.0203073.s004.pdf]

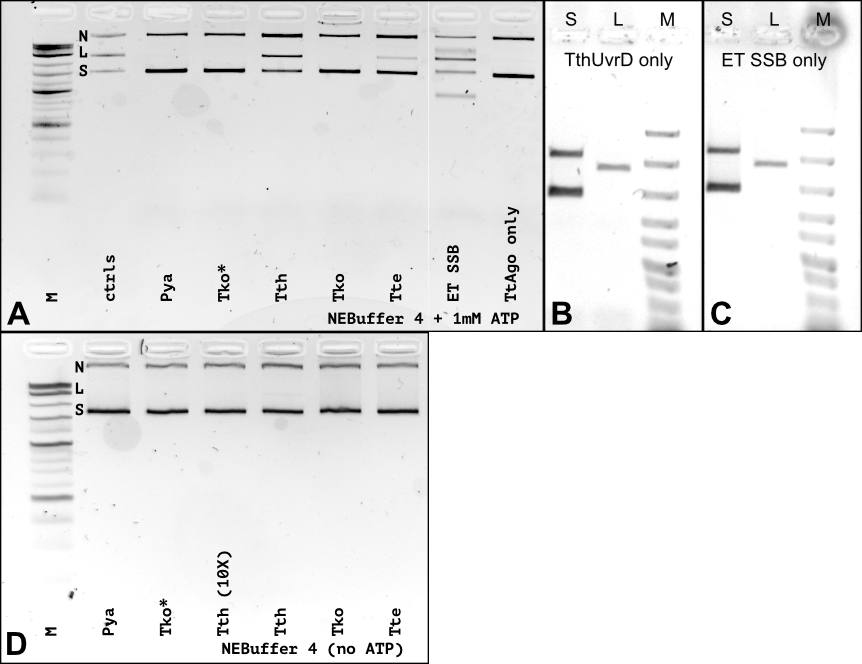

Supplement: S5 Fig — (A, D) Pya (Pyrococcus yayanosii); Tko (Thermococcus kodakarensis), the (*) signifies the sequence differs from the wildtype; Tth (Thermus thermophilus), (10X) refers to the helicase concentration compared to the following lane; Tte (Thermoanaerobacter tengcongensis). Reactions were performed in NEBuffer™ 4 with 1 mM ATP (A) or without ATP (D). Amount of helicase varied from 10–50ng per reaction. Guides were pre-loaded by incubation at 75 °C for 20 min followed by incubation for reaction at 73 °C for 4 hr. Reactions were halted by the addition of Proteinase K, incubated at 45 °C for 30 min. Guides were designed to target the BamHI site in supercoiled pUC19 substrate. Product was observed by the appearance of linearized pUC19 (A), but was not observed in the absence of ATP (D). For comparison ET SSB was also added under the same reaction conditions. Increased activity was observed with ET SSB which also showed some off-target cleavage generating a second product band. Increased activity was observed with the TthUvrD and perhaps some activity with the other bacterial TteUvrD, but not with the archaeal UvrD-like proteins. To verify that the cleavage products observed were not due to contamination in the TthUvrD or ET SSB preps, TthUvrD only (B) and ET SSB only (C) controls were carried out on supercoiled and linearized pBR322 plasmid at 73 °C for 8 hr. No degradation of either substrate was observed. Plasmid controls (ctrls) are nicked (N) with Nt.BspQI, linearized (L) with EcoRI, and supercoiled (S). The marker (M) is NEB Quick-Load Purple 2-Log DNA Ladder in (A, D), and NEB Fast DNA Ladder in (B, C). (JPG) [file pone.0203073.s005.jpg]

**A**

(no ETSSB - 5X Guide)

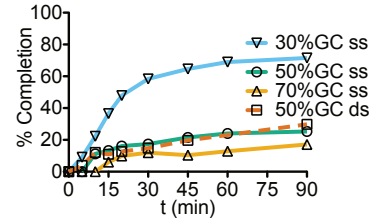**B**

5'FAM &amp; 3'TAM (0.5–10X Guide)

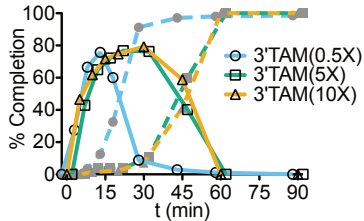**C**

5'FAM &amp; 3'TAM/5'HEX (10X Guide)

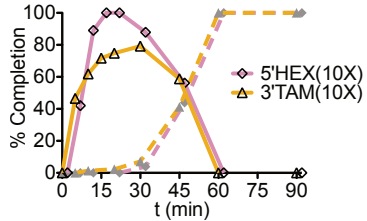

Supplement: S6 Fig — B) With an exposed 5′-phosphate present on the substrate, increasing guide concentration only marginally delays non-specific degradation of the substrate. C) Even in the absence of a 5′-phosphate and with excess guide, non-specific degradation of the substrate occurs in the presence of ET SSB. (PDF) [file pone.0203073.s006.pdf]

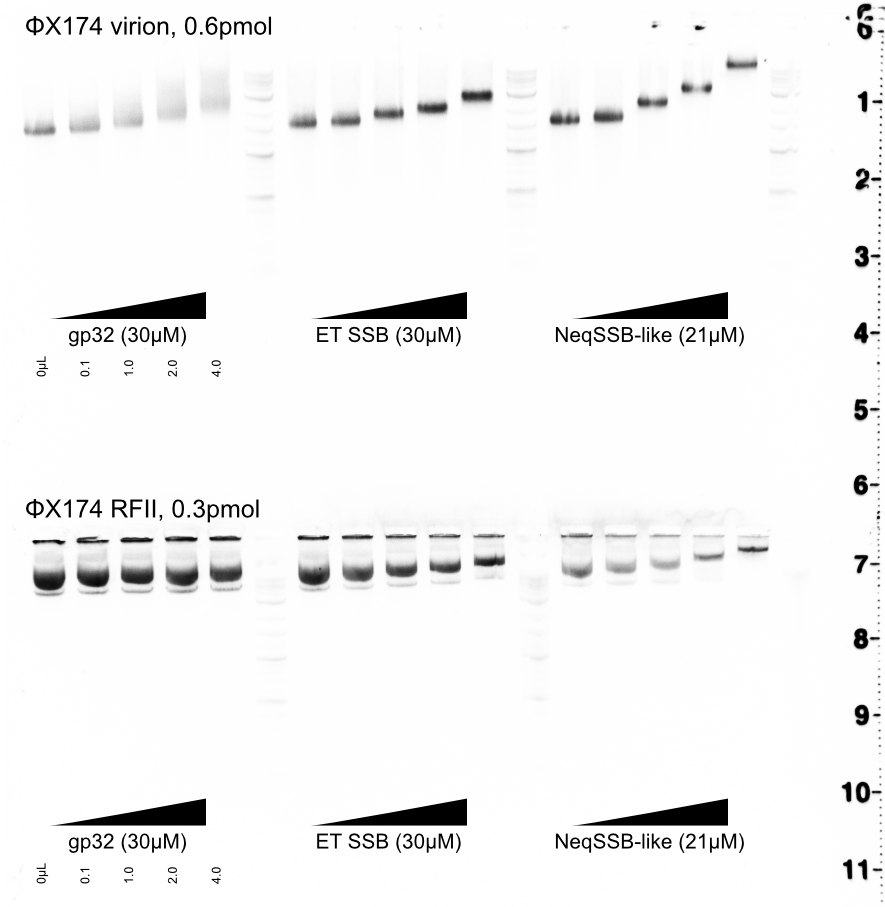

Supplement: S7 Fig — SSB binding activity for gp32, ET SSB, and NeqSSB-like protein observed by gel shift on 0.8% agarose TBE gel electrophoresis with ssDNA (ΦX174 virion) and relaxed, circular dsDNA (ΦX174 RFII). (PNG) [file pone.0203073.s007.png]

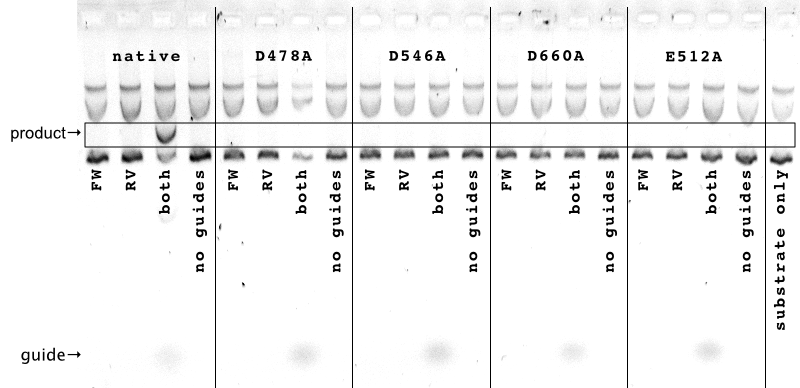

Supplement: S8 Fig — Point mutations D478A, E512A, D546A, and D660A were introduced separately using the Q5®Mutagenesis Kit (New England Biolabs) following the instructions provided with the kit. These four residues are known to be involved in the RNase H-type active site of TtAgo [20]. Mutants were prepared and purified according to the same expression and purification procedures provided in the Materials and methods section. Mutation of the active site eliminated endonuclease activity indicating that when taken in conjunction with the other controls and data provided in this manuscript, the activity observed with the non-mutated sequence can be genuinely accredited to TtAgo and not a contaminating nuclease. Reactions were carried out using guide set 5 as in Supporting information S1 Fig. (PNG) [file pone.0203073.s008.png]
