# Supplementary material for: Single-stranded binding proteins and helicase enhance the activity of prokaryotic argonautes in vitro
Source: PLoS One. 2018 Aug 29;13(8):e0203073. doi: 10.1371/journal.pone.0203073 (PMC6114923; doi:10.1371/journal.pone.0203073)
Supplement: S1 Table — Left→Right, 5′→3′; cleavage site between bases 10 and 11. (PDF) [file pone.0203073.s009.pdf]

S1 Table. Sequence and GC content information for guides. Left→Right, 5′→3′; cleavage site between bases 10 and 11.

Name	Sequence	Length	%GC
random sequence fully-complementary guides			
RAN30-FW_CUT	CAGTTATATTATCTGAGCACT	21	33%
RAN48-FW_CUT	CATACGACTGATCTGAGCACT	21	48%
RAN50-RV_CUT	CTGCTCAGATCAGTCGTATGT	21	48%
RAN67-FW_CUT	CAGTCGCGTGCCCTGAGCACT	21	67%
pUC19 guides			
pUC-1	GGATCCTCTAGAGTCGACCTG	21	62%
pUC-2	GGTCGACTCTAGAGGATCCCC	21	62%
ΦX174 guides			
ΦX174-1	CATACCAAAGACGAGCG	17	53%
ΦX174-2	CAACGGCTGCGGACGAC	17	71%
ΦX174-3	CATTACATCACTCCTTC	17	41%
ΦX174-4	CAAAGTCCAGCGTACCA	17	53%
ΦX174-5	CAACAAGAGAATCTCTA	17	35%
ΦX174-6	CAGTCCACTTCGATTTA	17	41%
random sequence mismatch guides			
RAN48-FW_CUT-A2G	CGTACGACTGATCTGAGCACT	21	52%
RAN48-FW_CUT-T3C	CACACGACTGATCTGAGCACT	21	52%
RAN48-FW_CUT-A4G	CATGCGACTGATCTGAGCACT	21	52%
RAN48-FW_CUT-C5T	CATATGACTGATCTGAGCACT	21	43%
RAN48-FW_CUT-G6A	CATACAACTGATCTGAGCACT	21	43%
RAN48-FW_CUT-A7G	CATACGGCTGATCTGAGCACT	21	52%
RAN48-FW_CUT-C8T	CATACGATTGATCTGAGCACT	21	43%
RAN48-FW_CUT-T9C	CATACGACCGATCTGAGCACT	21	52%
RAN48-FW_CUT-G10A	CATACGACTAATCTGAGCACT	21	43%
RAN48-FW_CUT-A11G	CATACGACTGGTCTGAGCACT	21	52%
RAN48-FW_CUT-T12C	CATACGACTGACCTGAGCACT	21	52%
RAN48-FW_CUT-C13T	CATACGACTGATTTGAGCACT	21	43%
RAN48-FW_CUT-T14C	CATACGACTGATCCGAGCACT	21	52%
RAN48-FW_CUT-G15A	CATACGACTGATCTAAGCACT	21	43%
RAN48-FW_CUT-A16G	CATACGACTGATCTGGGCACT	21	52%
RAN48-FW_CUT-G17A	CATACGACTGATCTGAACACT	21	43%
RAN48-FW_CUT-C18T	CATACGACTGATCTGAGTACT	21	43%
RAN48-FW_CUT-A19G	CATACGACTGATCTGAGCGCT	21	52%
RAN48-FW_CUT-C20T	CATACGACTGATCTGAGCATT	21	43%
RAN48-FW_CUT-T21C	CATACGACTGATCTGAGCACC	21	52%
random sequence variable length guides			
RAN48-FW_CUT-32	CATACGACTGATCTGAGCACTAGCTATAGCTA	32	44%
RAN48-FW_CUT-31	CATACGACTGATCTGAGCACTAGCTATAGCT	31	45%
RAN48-FW_CUT-30	CATACGACTGATCTGAGCACTAGCTATAGC	30	47%
RAN48-FW_CUT-29	CATACGACTGATCTGAGCACTAGCTATAG	29	45%
RAN48-FW_CUT-28	CATACGACTGATCTGAGCACTAGCTATA	28	43%
RAN48-FW_CUT-27	CATACGACTGATCTGAGCACTAGCTAT	27	44%
RAN48-FW_CUT-26	CATACGACTGATCTGAGCACTAGCTA	26	46%
RAN48-FW_CUT-25	CATACGACTGATCTGAGCACTAGCT	25	48%
RAN48-FW_CUT-24	CATACGACTGATCTGAGCACTAGC	24	50%
RAN48-FW_CUT-23	CATACGACTGATCTGAGCACTAG	23	48%
RAN48-FW_CUT-22	CATACGACTGATCTGAGCACTA	22	45%
RAN48-FW_CUT-20	CATACGACTGATCTGAGCAC	20	50%
RAN48-FW_CUT-19	CATACGACTGATCTGAGCA	19	47%
RAN48-FW_CUT-18	CATACGACTGATCTGAGC	18	50%
RAN48-FW_CUT-17	CATACGACTGATCTGAG	17	47%
RAN48-FW_CUT-16	CATACGACTGATCTGA	16	44%
RAN48-FW_CUT-15	CATACGACTGATCTG	15	47%
RAN48-FW_CUT-14	CATACGACTGATCT	14	43%
RAN48-FW_CUT-13	CATACGACTGATC	13	46%
RAN48-FW_CUT-12	CATACGACTGAT	12	42%
RAN48-FW_CUT-11	CATACGACTGA	11	45%
