# Supplementary material for: Single-stranded binding proteins and helicase enhance the activity of prokaryotic argonautes in vitro
Source: PLoS One. 2018 Aug 29;13(8):e0203073. doi: 10.1371/journal.pone.0203073 (PMC6114923; doi:10.1371/journal.pone.0203073)
Supplement: S2 Table — (PDF) [file pone.0203073.s010.pdf]

S2 Table. Sequence and GC content information for CE substrates.

Name	Sequence	Length	%GC
ssDNA			
FAM-RAN30_98FW	/56-FAM/<br>ATCATATTGGAGTTGAATGTAGGTTATAATAATATTTACT<br>AAGCGACTAAGTGCTCAGATAATATAACTGTAGCTTAAGC<br>TTATTTACCCTACACTAT	98	30
FAM-RAN49_98FW	/56-FAM/<br>CTCTACATTCTGGTGCAGCCCGATAAAGCCTACTGAGGGT<br>TAAATAGCTAGTGCTCAGATCAGTCGTATGTAGGCTTGTG<br>CACACCATTAGCGGTAGC	98	49
FAM-RAN67_98FW	/56-FAM/<br>AACACCAGGGAGCTCCTGCGCTTGCCTAACGGGATATGCG<br>CGACCGGCCAGTGCTCAGGGCACGCGACTGGTGGTCCGGC<br>CAGCCGTCGTTGCTGGTC	98	67
dsDNA			
FW5FAM-RV5HEX_RAN30	/56-FAM/<br>ATCATATTGGAGTTGAATGTAGGTTATAATAATATTTACT<br>AAGCGACTAAGTGCTCAGATAATATAACTGTAGCTTAAGC<br>TTATTTACCCTACACTAT<br><br>/5HEX/<br>ATAGTGTAGGGTAAATAAGCTTAAGCTACAGTTATATTAT<br>CTGAGCACTTAGTCGCTTAGTAAATATTATTATAACCTAC<br>ATTCAACTCCAATATGAT	98	30
FW5FAM-RV3TAM_RAN48	/56-FAM/<br>ATCTACATTCTGGTGCAGCCCGATAAAGCCTACTGAGGGT<br>TAAATAGCTAGTGCTCAGATCAGTCGTATGTAGGCTTGTG<br>CACACCATTAGCGGTAGC<br><br>GCTACCGCTAATGGTGTGCACAAGCCTACATACGACTGAT<br>CTGAGCACTAGCTATTTAACCCTCAGTAGGCTTTATCGGG<br>CTGCACCAGAATGTAGAT<br>/36-TAMSp/	98	48
FW5FAM-RV5HEX_RAN48	/56-FAM/<br>CTCTACATTCTGGTGCAGCCCGATAAAGCCTACTGAGGGT<br>TAAATAGCTAGTGCTCAGATCAGTCGTATGTAGGCTTGTG<br>CACACCATTAGCGGTAGT<br><br>/5HEX/<br>ACTACCGCTAATGGTGTGCACAAGCCTACATACGACTGAT<br>CTGAGCACTAGCTATTTAACCCTCAGTAGGCTTTATCGGG<br>CTGCACCAGAATGTAGAG	98	48
FW5FAM-RV5HEX_RAN67	/56-FAM/<br>AACACCAGGGAGCTCCTGCGCTTGCCTAACGGGATATGCG<br>CGACCGGCCAGTGCTCAGGGCACGCGACTGGTGGTCCGGC<br>CAGCCGTCGTTGCTGGCT<br><br>/5HEX/<br>AGCCAGCAACGACGGCTGGCCGGACCACCAGTCGCGTGCC<br>CTGAGCACTGGCCGGTCGCGCATATCCCGTTAGGCAAGCG<br>CAGGAGCTCCCTGGTGTT	98	67
ssRNA & dsRNA			
FW5FAM_rRAN45	/56-FAM/<br>CGAUAAAGCCUACUGAGGGUUAAAUAGCUAGUGCUCAGAU<br>CAGUCGUAUGUAGGCUUGUG	60	45
FW5FAM_rRAN45dup	*same as above, duplexed with antisense*		
