# Supplementary material for: Single-stranded binding proteins and helicase enhance the activity of prokaryotic argonautes in vitro
Source: PLoS One. 2018 Aug 29;13(8):e0203073. doi: 10.1371/journal.pone.0203073 (PMC6114923; doi:10.1371/journal.pone.0203073)
Supplement: S1 Appendix — ET SSB did not improve NgAgo activity as a DNA-guided DNA or RNA endonuclease. (PDF) [file pone.0203073.s011.pdf]

S1 Appendix.

Thermostable SSB did not improve *Ng*Ago activity.

Significant attention greeted the publication by Gao et al. [1] describing the use of *Ng*Ago as a DNA-guided DNA endonuclease for genome editing applications, but the recent retraction of the publication along with the many difficulties reported in reproducing the work described therein [2, 3] has added a great deal of skepticism to these claims. We produced and purified *Ng*Ago according to the methods of Sunghyeok et al. [4] and investigated whether thermostable SSBs could improve or impart DNA endonuclease activity. We were able to observe some specific DNA-guided RNA endonuclease activity as described by Sunghyeok et al. using the same cleavage conditions, CE substrates, and CE analysis protocols used for *Tt*Ago, however the addition of ET SSB and *Neq*SSB-like protein did not have an effect on activity. We note that the expression and purification of active *Ng*Ago was not as straightforward as described by Sunghyeok et al., and activity was only observed when purified under denaturing conditions (see below). While endonuclease activity on dsDNA at moderate temperature would be a significant addition to the genome editing toolbox, our attempts to improve its activity as observed for other pAgos were unsuccessful. We feel that more evidence is still needed to verify the results of *Ng*Ago functioning as a DNA-guided RNA endonuclease.

Expression and Purification of *Ng*Ago

*Ng*Ago was expressed and purified according to both methods described by Sunghyeok et al. [4]. DNA-guided RNA endonuclease activity was only observed when *Ng*Ago was purified by IMAC FPLC using a 5mL HisTrap FF column on an KTA FPLC (GE Healthcare Life Sciences) under denaturing conditions with 6 M guanidine hydrochloride. Purified *Ng*Ago was dialyzed into NEB diluent E (10 mM Tris-HCl pH 7.4 @ 25 ℃, 100 mM potassium chloride; 1 mM DTT, 0.1 mM EDTA, 50% glycerol) and stored at -20 ℃.

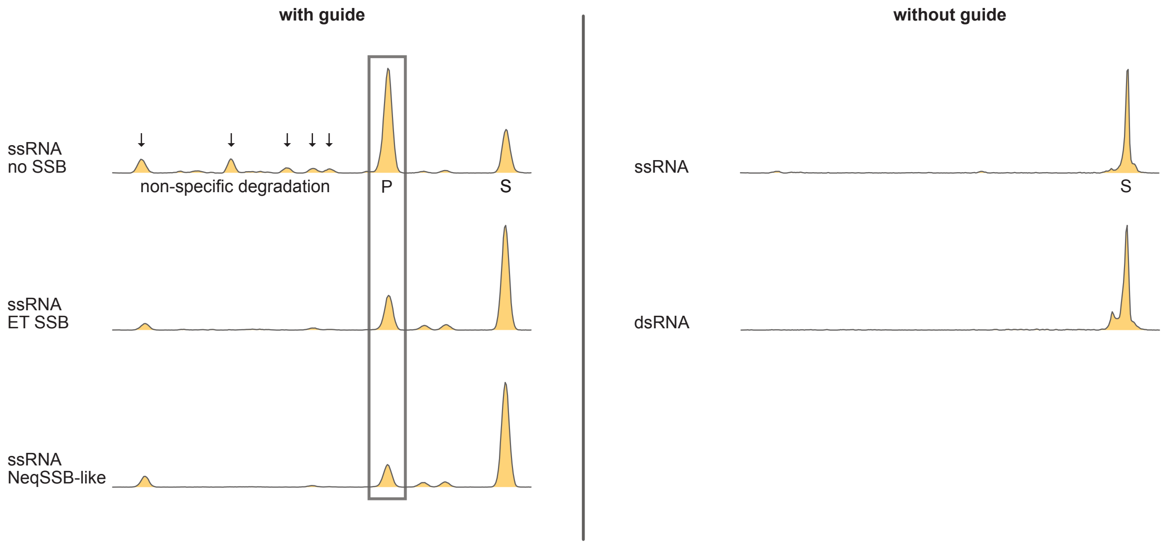

*Ng*Ago was incubated at 37 ℃ for 12 hr *with* 5′-phosphorylated DNA guide and ssRNA 5′ FAM-labeled substrate (left). Full-length substrate (60 nt) is marked with (**S**) and product (40 nt) is marked with (**P**). Neither ET SSB nor *Neq*SSB-like protein improved *Ng*Ago DNA-guided RNA endonuclease activity. As a control to ensure the cleavage product observed was not due to contaminating RNase from the prep, *Ng*Ago was incubated at 37 ℃ for 12 hr *without* 5′-phosphorylated DNA guide (right) in the presence of ssRNA or dsRNA 5′ FAM-labeled substrate. No degradation was observed following this incubation with apo-*Ng*Ago suggesting this result was not due to a contaminating endonuclease.

[1] Gao F, Shen XZ, Jiang F, Wu Y, Han C. DNA-guided genome editing using the Natronobacterium gregoryi Argonaute. Nat Biotechnol. 2016;34(7):768–773.

[2] Lee SH, Turchiano G, Ata H, Nowsheen S, Romito M, Lou Z, et al. Failure to detect DNA-guided genome editing using Natronobacterium gregoryi Argonaute. Nat Biotechnol. 2016;35(1):17–18.

[3] Javidi-Parsijani P, Niu G, Davis M, Lu P, Atala A, Lu B. No evidence of genome editing activity from Natronobacterium gregoryi Argonaute (NgAgo) in human cells. PLoS One. 2017;12(5):e0177444.

[4] Sunghyeok Y, Taegeun B, Kyoungmi K, Omer H, Seung Hwan L, Yoon Young K, et al.. DNA-dependent RNA cleavage by the Natronobacterium gregoryi Argonaute; 2017.
